# Secreted ORF8 reprograms macrophages to enhance SARS-CoV-2 infection of lung epithelial cells

**DOI:** 10.1126/sciadv.aee5421

**Published:** 2026-09-18

**Authors:** Yusuke Matsui, Rahul K. Suryawanshi, Mauricio Montano, Taha Y. Taha, Mir M. Khalid, Limeng Sun, Kanika Khanna, Jin Tang, Yuan Zhou, Robyn M. Kaake, Xiaohui Fang, Mazharul Maishan, Michael A. Matthay, Nevan J. Krogan, Melanie Ott

**Affiliations:** ^1^ J. David Gladstone Institutes; San Francisco, CA, USA.; ^2^ Michael Hulton Center for HIV Cure Research at Gladstone; San Francisco, CA, USA.; ^3^ Laboratory of Neurological Infections and Immunity, Division of Intramural Research, National Institute of Allergy and Infectious Diseases, Rocky Mountain Laboratories; Hamilton, MT, USA.; ^4^ Department of Bioengineering and Therapeutic Sciences, University of California San Francisco; San Francisco, CA, USA.; ^5^ Quantitative Biosciences Institute (QBI), University of California San Francisco; San Francisco, CA, USA.; ^6^ Cardiovascular Research Institute, University of California San Francisco; San Francisco, CA, USA.; ^7^ Department of Anesthesia, University of California San Francisco; San Francisco, CA, USA.; ^8^ Department of Medicine, University of California San Francisco; San Francisco, CA, USA.; ^9^ Biohub; San Francisco, CA, USA.

## Abstract

Severe acute respiratory syndrome coronavirus 2 (SARS-CoV-2) primarily targets the respiratory epithelium, yet severe disease features diffuse lung injury and hyperinflammatory syndromes driven by dysregulated immune activation. Emerging evidence indicates that resident and infiltrating immune cells in the lung can encounter the virus early in infection and, under specific conditions, become infected. This process amplifies local inflammation and facilitates viral propagation in the lower airways and distal lung regions, where angiotensin converting enzyme 2 (ACE2) expression is limited. However, how epithelial and immune cell compartments interact to produce the hallmark pulmonary pathology of SARS-CoV-2 infection remains unresolved. Here we show that secreted ORF8, a SARS-CoV-2 accessory protein, drives inflammatory lung pathology by increasing macrophage permissiveness to infection, triggering pyroptosis, and amplifying viral replication in alveolar epithelial cells. Co-culture of macrophages with human alveolar type II (AT2) cells overrides ORF8’s previously reported inhibition of AT2 infection, restoring infectious viral production. In vivo, IL-17RA blockade counteracts ORF8 activity, lowering viral burden and attenuating pulmonary inflammation and fibrosis. These findings reveal a paracrine role for ORF8 in reprogramming macrophages, thereby establishing a feedforward proviral circuit that accelerates lung pathology in COVID-19 and are clinically relevant given the recurrent emergence of SARS-CoV-2 variants with either intact or deleted ORF8 since the beginning of the pandemic.

## INTRODUCTION

SARS-CoV-2 continues to circulate worldwide despite widespread vaccination and immunity acquired through prior infection (*1*, *2*). Within the *Betacoronavirus* genus, the open reading frame 8 (ORF8) is among the most variable genomic regions, frequently undergoing recombination events (*3*–*6*). ORF8 encodes a secreted accessory protein linked to viral pathogenicity, and serum ORF8 levels in patients with COVID-19 correlate with disease severity (*6*–*11*). Variants lacking ORF8, such as the Δ382 strain identified in Singapore, are associated with attenuated disease and reduced hypoxia compared to wild-type virus (*12*–*15*). In vitro, ORF8 has been reported to suppress type I interferon signaling, downregulate MHC-I expression, and disrupt host epigenetic control via histone H3 mimicry (*16*, *17*). Conversely, ORF8 has also been shown to interfere with viral assembly (*18*, *19*), limit new virion production, and bind extracellularly to interleukin-17 receptor A (IL-17RA) (*20*), a mediator of pro-inflammatory signaling. These diverse and sometimes contradictory findings leave the contribution of ORF8 to viral spread and immunopathology mostly unresolved.

Macrophages are central sentinels of the respiratory immune barrier and play a pivotal role in shaping the host response to SARS-CoV-2 (*21*). Severe COVID-19 is characterized by macrophage-driven hyperinflammation (*22*–*29*), with increased infiltration of macrophages into the lungs (*30*, *31*) and genetic associations link inflammasome and pyroptosis pathways to disease severity (*32*). Previous studies have demonstrated that blood monocytes and pulmonary macrophages can be infected by SARS-CoV-2, leading to pyroptotic cell death and increased production of inflammatory cytokines (*25*, *33*). However, whether macrophages serve merely as amplifiers of inflammation or as direct targets of productive infection remains a matter of ongoing debate (*25*, *34*–*37*). Here, we tested the hypothesis that ORF8 modified the function of macrophages and their sensitivity to SARS-CoV-2 viral infection.

## RESULTS

### Soluble ORF8 protein enhances the uptake of SARS-CoV-2 VLPs by macrophages

To investigate the effect of extracellular ORF8 on SARS-CoV-2 entry into macrophages, we incubated macrophages with virus-like particles (VLPs) (*38*) that incorporated SARS-CoV-2 Spike, Nucleocapsid, E protein, M protein, and the firefly luciferase mRNA (Fig. 1A). We found that primary monocyte-derived macrophages (MDMs) take up VLPs irrespective of their polarization into unstimulated macrophages (M0), classically activated macrophages (M1), or alternatively activated macrophages (M2) (fig. S1A). Although VLP-derived luciferase signals in MDMs were substantially lower than those observed in 293 T-ACE2/TMPRSS2 cells (fig. S2A), exogenous ORF8 enhanced VLP entry into all MDM subsets in a concentration-dependent manner, with maximal enhancement observed at 1 μg/mL (Fig. 1B). Previous studies have reported circulating ORF8 concentrations of approximately 400 ng/mL in patients with severe COVID-19 (*11*); however, the local concentration of ORF8 within infected tissue microenvironments remains unknown. Therefore, we examined a broad concentration range of recombinant ORF8 (0.1–3 μg/mL) in our in vitro assays.

**Fig. 1.**
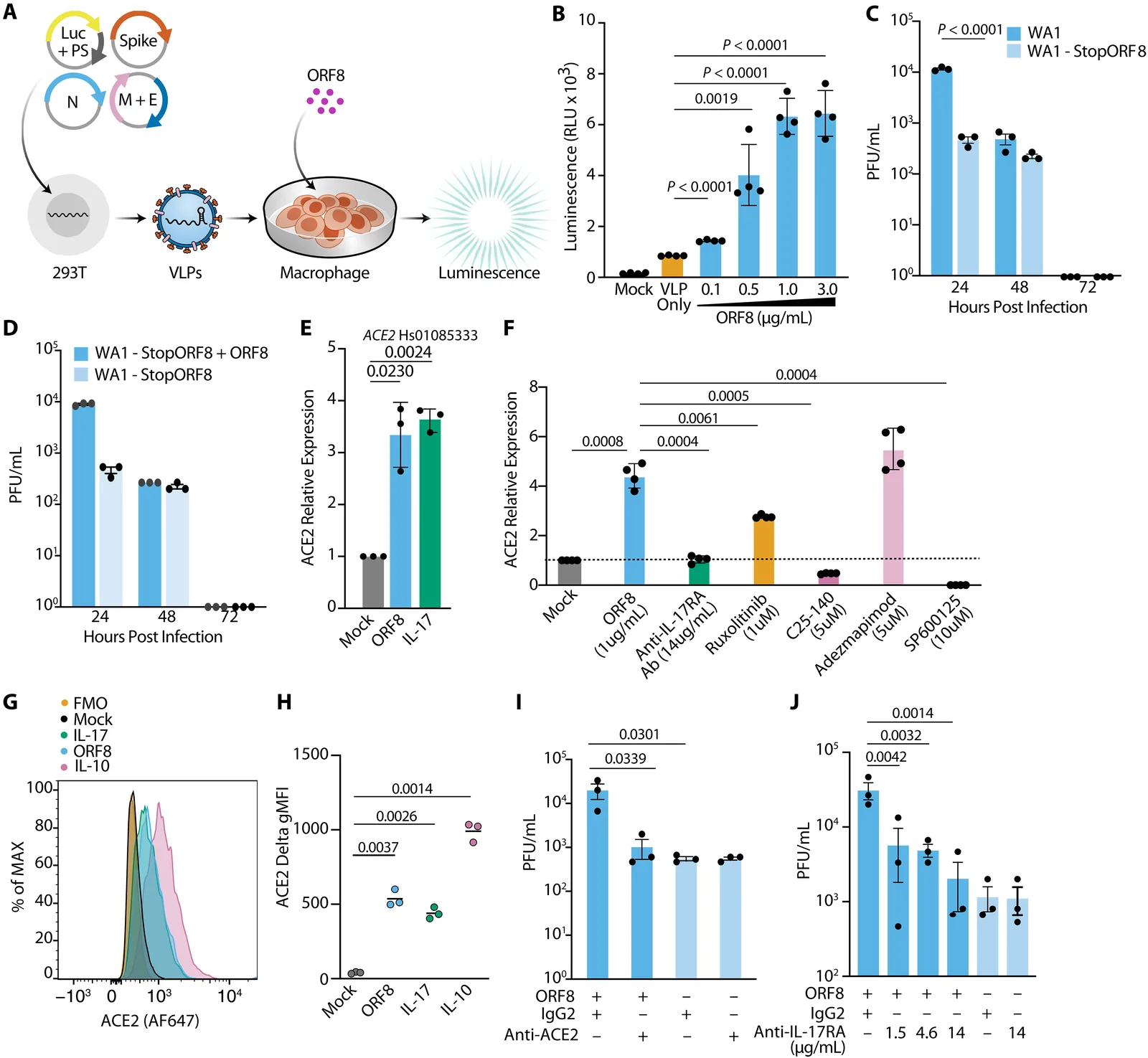
VLP and virion uptake into macrophages is enhanced by soluble ORF8 protein. (**A**) Schematic of VLP generation and infection of primary monocyte-derived macrophages (MDMs) in the presence of extracellular ORF8. Created in BioRender. Matsui, Y. (2026) https://biorender.com/yssgap0 (**B**) MDMs were treated with increasing concentrations of ORF8 for 48 h and infected with luciferase-expressing SARS-CoV-2 VLPs. Luciferase activity was measured 24 h later. (**C** and **D**) MDMs were infected with SARS-CoV-2 WA1 or WA1-StopORF8 (C), or WA1-StopORF8 in the presence or absence of recombinant ORF8 (D). Infectious virus released into the culture supernatants was quantified by plaque assay and expressed as plaque-forming units (PFU)/mL at the indiacated time points. (**E**) Expression of the indicated genes in MDMs treated with ORF8 or IL-17 was quantified by qPCR. (**F**) MDMs were pretreated with brodalumab (anti–IL-17RA), ruxolitinib, C25–140, adezmapimod, or SP600125 prior to ORF8 stimulation, and ACE2 mRNA expression was measured by qPCR. (**G** and **H**) Surface ACE2 expression was quantified by flow cytometry following treatment with ORF8, IL-17, or IL-10. DeltaMFI was calculated by subtracting fluorescence-minus-one (FMO) control values from stained samples. (**I** and **J**) MDMs were treated with ORF8 alone or in combination with anti-ACE2 antibody (I) or brodalumab (J), followed by infection with WA1-StopORF8. Culture supernatants were analyzed by plaque assay and viral titers expressed as PFU/mL 24 h after infection. *n* = 3, except (B) and (F) (*n* = 4). Statistical significance was determined by one-way ANOVA with Kruskal-Wallis and Dunn’s multiple-comparison tests [(B), (E) to (J)] or two-tailed Welch’s *t* test [(C) and (D)].

### Virally-produced or externally provided ORF8 transiently increases infectious viral titers released from infected macrophages

To assess whether infected macrophages can produce infectious viurs, we first exposed MDMs to either a SARS-CoV-2 WA1 replicon containing a luciferase reporter gene or to replication-competent SARS-CoV-2 WA1. In the replicon system (*39*). luminescence revealed a significant increase in RNA replication 24 h postinfection. (fig. S1C). In infections with the replication-competent virus, the presence of double-stranded RNA (dsRNA) was detected inside the macrophages by immunostaining as early as 10 h postinfection, and of nucleocapsid protein by 24 h (fig. S1D). We next asked whether virally-produced ORF8 influences the production of infectious virus. To this end, we infected MDMs with either the replication-competent SARS-CoV-2 WA1 or a mutant strain with a stop codon inserted in the ORF8 gene sequence (StopORF8) at a multiplicity of infection (MOI) of 2 and measured the infectious viral titers released in the supernatant via a plaque assay. At 24 h postinfection, the wild-type virus expressing ORF8 produced 10 times more infectious viral titers than the StopORF8 virus (Fig. 1C). This increase in infectious viral titers rapidly declined after 48 h postinfection and by 72 h postinfection, infectious virus production in both WA1- and WA1-StopORF8-infected cells completely ceased (Fig. 1C). To test whether externally provided ORF8 could also increase viral production, MDMs were infected with WA1-StopORF8 in the presence of 1 μg/mL full-length extracellular ORF8. Under these conditions, infectious viral titers at 24 h postinfection increased approximately 10-fold compared with WA1-StopORF8 infection alone, reaching levels comparable to those observed with wild-type WA1 infection (Fig. 1D). To evaluate whether ORF8-dependent enhancement of infectious viral production could also be observed in a naturally occurring ORF8-deficient SARS-CoV-2 variant, we repeated these experiments with the XBB.1.5 strain, which carries a G8 nonsense mutation in ORF8 (*40*), in the presence or absence of 1 μg/mL extracellular ORF8. The addition of soluble ORF8 resulted in higher viral titers up to at least 48 h postinfection, but by 72 h postinfection, infectious virus production was undetectable regardless of the presence or absence of extracellular ORF8. (fig. S1E).

### Extracellular ORF8 upregulates ACE2 expression on the macrophages’ cell surface via IL-17RA

To elucidate the mechanism by which ORF8 promotes virion entry into macrophages, we treated MDMs with ORF8 and then measured candidate entry pathway genes by RT-qPCR (Fig. 1E and fig. S1F). Extracellular ORF8 treatment induced statistically significant increases in expression of the viral receptor gene *ACE2* (angiotensin-converting enzyme 2) (*41*) (Fig. 1E). To identify signaling pathways involved in ORF8-mediated activation of ACE2 transcription in MDMs, cells were stimulated with extracellular ORF8 for 3 h followed by RNA-seq analysis (fig. S2, A and B). Transcriptomic profiling revealed robust induction of inflammatory and chemotactic programs strongly associated with NF-κB and AP-1 signaling. Among the most highly upregulated genes were canonical NF-κB target genes, including IL1B, TNF, CXCL1, CXCL2, CXCL3, CXCL8, CCL3, CCL4, CCL2, TRAF1, PTX3, and TNFAIP6, consistent with activation of a broad proinflammatory transcriptional response. In parallel, multiple negative-feedback regulators of NF-κB signaling, including NFKBIA, NFKBIZ, ZC3H12A, and IER3, were also induced, suggesting engagement of compensatory mechanisms that limit excessive inflammatory activation (fig. S2A). The induction of EDN1, OSM, JAG1, and G0S2 further supported activation of stress-responsive transcriptional programs downstream of NF-κB/AP-1 signaling. Notably, these transcriptional features were also consistent with activation of IL-17-associated signaling pathways (fig. S2B). Collectively, these findings indicate that ORF8 rapidly activates innate immune programs in human macrophages characterized by coordinated induction of inflammatory cytokines, chemokines, and NF-κB/AP-1-associated regulatory networks. To further define the temporal dynamics of NF-κB and AP-1 activation induced by ORF8, we performed NanoLuc reporter assays in MDMs transduced with NF-κB- or AP-1-responsive reporter lentiviruses (fig. S2, C and D). Cells were stimulated with ORF8 (1 μg/mL), IL-17 (100 ng/mL), ORF8 plus IL-17, or positive controls consisting of LPS (100 ng/mL) for NF-κB reporter assays and PMA (40 nM) for AP-1 reporter assays. Culture supernatants were collected at 5, 10, and 24 h post-stimulation, and extracellular NanoLuc activity was quantified. NF-κB reporter activity in ORF8-stimulated cells was rapidly induced at 5 h but declined to near-baseline levels by 24 h. IL-17 stimulation produced a similar temporal profile. Notably, combined stimulation with ORF8 and IL-17 resulted in strong early NF-κB activation at 5 h, followed by a rapid decline at 10 h and near-complete resolution by 24 h (fig. S2C). In contrast, AP-1 reporter activity exhibited a distinct kinetic profile. Both ORF8 and IL-17 induced relatively modest AP-1 activation at 5 h, which progressively increased over time and reached maximal levels at 24 h (fig. S2D). Co-stimulation with ORF8 and IL-17 similarly resulted in sustained AP-1 activation without the decline observed in NF-κB signaling. Collectively, these findings suggest that ORF8 induces transient NF-κB activation but sustained AP-1 signaling in human macrophages. Building on these findings, we next examined whether ORF8-induced ACE2 expression was functionally linked to IL-17-associated inflammatory signaling pathways (Fig. 1F). MDMs were stimulated with recombinant ORF8 (1 μg/mL) in the presence or absence of an anti-IL-17RA antibody (brodalumab), the JAK–STAT inhibitor ruxolitinib, the TRAF6 inhibitor C25–140, the p38 inhibitor adezmapimod, or the JNK inhibitor SP600125, followed by quantification of ACE2 mRNA expression. ORF8-induced ACE2 expression was strongly suppressed by IL-17RA blockade, TRAF6 inhibition, and JNK inhibition, whereas JAK–STAT inhibition produced a more modest reduction (Fig. 2F). These findings demonstrate that ORF8 promotes ACE2 transcription primarily through an IL-17RA–TRAF6–JNK signaling axis, consistent with sustained AP-1 activation observed in the reporter assays (fig. S2D). Flow cytometric analysis demonstrated that ORF8-treated MDMs exhibited increased ACE2 surface expression compared with untreated controls (Fig. 1, G and H). As a positive control, we also confirmed the previously reported IL-10-mediated upregulation of ACE2 expression (*42*). Since ORF8 interacts with the IL-17RA receptor (*11*, *20*), we also incubated MDMs with IL-17. This treatment led to an increase in *ACE2* mRNA expression (Fig. 1E) as well as elevated ACE2 protein levels on the cell surface (Fig. 1, G and H), mirroring the effects observed with extracellular ORF8.

**Fig. 2.**
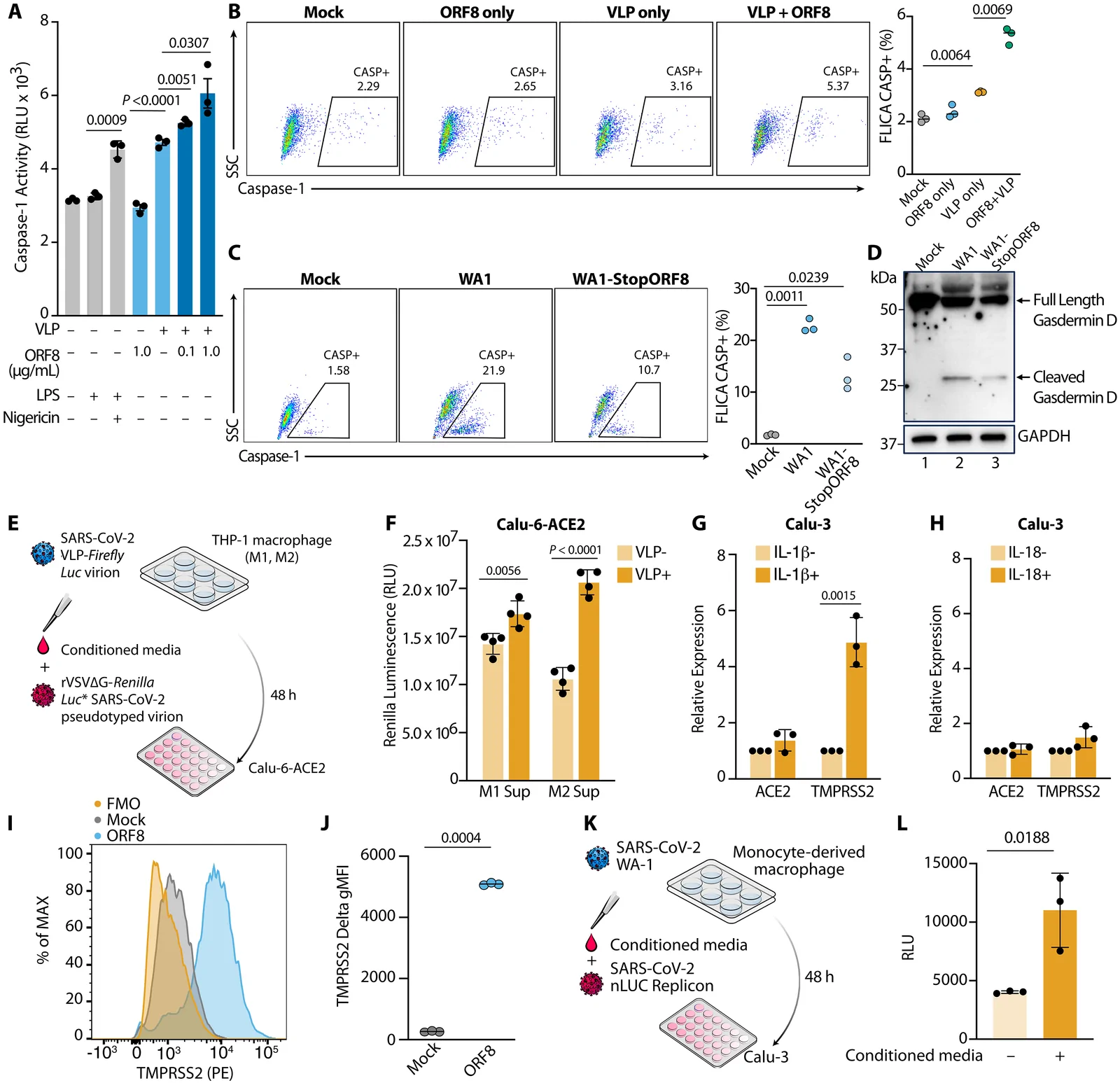
Extracellular ORF8 together with VLP uptake induces pyroptosis in macrophages. (**A**) MDMs were treated with ORF8, VLPs, or both, and extracellular caspase-1 activity was measured. LPS plus nigericin served as a positive control. (B) Caspase-1 activation in MDMs exposed to VLPs with or without ORF8 was quantified by flow cytometry using FAM-YVAD-FMK staining. Representative plots and summary data are shown. (**C**) Caspase-1 activation in MDMs infected with SARS-CoV-2 WA1 or WA1-StopORF8. (**D**) Western blot analysis of adherent and non-adherent cells following infection with WA1 or WA1-StopORF8. (**E** and **F**) Schematic and results of a macrophage-to-epithelial signaling assay. THP-1–derived macrophages were exposed to SARS-CoV-2 VLPs, and cell-free conditioned media were transferred to Calu-6-ACE2 cells together with SARS-CoV-2 Spike–pseudotyped reporter virions to assess epithelial cell susceptibility to viral entry. (**G** and **H**) Expression of the indicated genes in Calu-3 cells treated with IL-1β or IL-18 was quantified by RT-qPCR. (**I** and **J**) TMPRSS2 surface expression in Calu-3 cells following IL-1β treatment was measured by flow cytometry. (**K** and **L**) MDMs were infected with replication-competent SARS-CoV-2 WA1, and cell-free conditioned media were transferred to Calu-3 cells together with a SARS-CoV-2 NanoLuc replicon to evaluate epithelial cell susceptibility. *n* = 3 unless otherwise indicated; *n* = 4 for (F). Statistical significance was determined by one-way ANOVA with Kruskal-Wallis testing (A) or two-tailed Student’s *t* test [(B), (G) to (L)]. (E) and (K) created in BioRender. Matsui, Y. (2026) https://biorender.com/dqfdw8r.

To determine whether ORF8-mediated enhancement of ACE2 expression is essential for SARS-CoV-2 infection of macrophages, we assessed de novo viral production of WA1-StopORF8 in the presence or absence of extracellular ORF8, with or without anti-ACE2 antibody AF933, which has previously been reported to inhibit ACE2-dependent viral entry (*43*–*45*). In the absence of extracellular ORF8, treatment with the anti-ACE2 antibody had no significant effect on infectious viral titers. By contrast, in the presence of extracellular ORF8, anti-ACE2 antibody treatment significantly reduced viral production compared with no-antibody controls, decreasing it to levels comparable to those observed in the absence of extracellular ORF8 (Fig. 1I). Using the same approach, we next examined whether extracellular ORF8 promotes macrophage infection through the IL-17RA receptor. The addition of brodalumab (anti-IL-17RA antibody) inhibited extracellular ORF8–mediated enhancement of infectious viral titers in macrophages in a concentration-dependent manner (Fig. 1J). These findings indicate that extracellular ORF8 promotes the entry of both VLPs and virions into macrophages by upregulating ACE2 expression on the macrophage cell surface via IL-17RA.

### Extracellular ORF8 combined with VLP uptake induces pyroptosis in macrophages

The drastic decrease in infectious viral titers 48 h after infection led us to hypothesize that ORF8-assisted infection induces pyroptosis in macrophages. To test this hypothesis and assess whether ORF8 influences pyroptosis, we evaluated caspase-1 activity (*46*) in the supernatants of MDMs exposed to VLPs with or without soluble ORF8. Whereas ORF8 alone did not activate caspase-1, the combination of VLPs and ORF8 strongly induced caspase-1 activation (Fig. 2A). Furthermore, ORF8 enhanced caspase-1 activity in a dose-dependent manner, with stronger activation observed at 1 μg/mL compared with 0.1 μg/mL ORF8. Increased extracellular caspase-1 activity was used as a surrogate marker of inflammasome-associated pyroptotic cell death, suggesting activation of inflammasome-mediated pyroptotic pathways in macrophages following ORF8-assisted viral uptake. To quantify this effect, we labeled activated caspase-1 using FAM-YVAD-FMK (FLICA), a fluorescent inhibitor probe that covalently binds to active caspse-1, and quantified live caspase-1-activated cells by flow cytometry. The addition of VLPs and ORF8 to MDMs increased the percentage of caspase-1-activated cells by 1.5-fold compared to VLPs alone (Fig. 2B). These findings indicate that while extracellular ORF8 by itself does not trigger pyroptosis in macrophages, in combination with VLPs it enhances VLP uptake by macrophage, thereby lowering the threshold for pyroptosis. In a similar experiment using the replication-competent SARS-CoV-2 strains WA1 and WA1-StopORF8, infection with WA1 induced approximately twofold more caspase-1-activated cells than infection with WA1-StopORF8 (Fig. 2C). To further assess pyroptotic cell death, both adherent cells and floating cells in the culture supernatant were collected after overnight infection and analyzed by Western blotting. Cleaved gasdermin D was more prominently detected in WA1-infected samples than in WA1-StopORF8-infected samples, further supporting enhanced pyroptotic signaling mediated by ORF8 (Fig. 2D).

### IL-1β, a product of macrophage pyroptosis, upregulates TMPRSS2 in lung epithelial cells

In severe SARS-CoV-2 lung lesions, cytokine activation is amplified through cross-talk between epithelial and resident immune cells, with pyroptotic death of bystander myeloid cells proposed to drive cytokine production by epithelial cells (*47*, *48*). We aimed to define the mechanistic significance of macrophage pyroptosis, potentiated by ORF8 during SARS-CoV-2 infection, in shaping epithelial cell pathology within the lung. To assess the potential impact of pyroptosis following macrophage infection, we exposed a lung epithelial cell line (Calu-6-ACE2) to the supernatant of infected macrophages before infecting it with SARS-CoV-2 pseudotyped virions. THP-1-derived macrophages were exposed to the VLP described above harboring firefly luciferase mRNA with or without the addition of soluble ORF8, and after 48 h, the macrophage supernatant was mixed with rVSVΔG-Renilla luciferase SARS-CoV-2 pseudotyped virions and applied to Calu-6-ACE2 cells. To exclude potential signal contamination arising from carryover of the original firefly luciferase-containing VLPs present in the conditioned media, pseudotyped virions carrying a distinct Renilla luciferase reporter were intentionally used. Renilla luminescence was quantified 24 h later as a measure of pseudotyped virion entry (Fig. 2E). Supernatant from both classically activated macrophages (referred to as the M1phenotype) and alternatively activated macrophages (M2) infected by the SARS-CoV-2 VLP, enhanced virion entry into Calu-6-ACE2 cells (Fig. 2F). To test whether early cytokines IL-1β and IL-18 mediate macrophage-driven remodeling of epithelial surface receptor expression, we treated Calu-3 cells with IL-1β or IL-18 (caspase-1-matured). IL-18 had no effect on ACE2 or TMPRSS2 mRNA, while IL-1β significantly upregulated TMPRSS2 transcripts compared with vehicle (Fig. 2, G and H). Furthermore, flow cytometric analysis of IL-1β-stimulated Calu-3 cells demonstrated increased TMPRSS2 surface expression (Fig. 2I). Calu-3 were used after culture on Transwell inserts under air-liquid interface conditions to induce epithelial polarization (*49*). To confirm the infection-enhancing effect of supernatants derived from infected macrophages on lung epithelial cells, MDMs were exposed to replication-competent SARS-CoV-2 WA1, followed by extensive washing to remove residual input virus. Culture supernatants were collected 48 h postinfection and added to Calu-3 cells together with a SARS-CoV-2 replicon carrying a NanoLuc reporter. NanoLuc activity measured 24 h later revealed significantly higher relative luminescence units in cells treated with conditioned media from infected macrophages, indicating enhanced viral replication in epithelial cells (Fig. 2, K and L).

### Co-culture with macrophages allows the replication of ORF8-expressing SARS-CoV-2 in AT2 lung epithelial cells

To investigate epithelial cells-macrophage interaction during SARS-CoV-2 infection and tease apart the role of ORF8 under more physiological conditions, we infected mono- and co-cultures of MDMs and primary lung epithelial cells with WA1 or WA1-stopORF8 viruses. Initially, MDMs were spread at the bottom of the Transwell plates, and the virus was added at an MOI of 1. Viral mRNA was consistently higher in WA1-infected cells than in WA1-stopORF8-infected cells, supporting a proviral role of ORF8 in macrophage infection (Fig. 3A). Plaque assays further demonstrated that infectious virus was detectable only in the supernatants of WA1-infected cultures at 24 h postinfection, whereas infectious virus was undetectable in WA1-StopORF8-infected cultures at 24, 48, and 72 h postinfection (Fig. 3A). In contrast, in monocultures of primary alveolar type 2 (AT2) cells, infection with WA1-stopORF8 resulted in higher levels of viral RNA than infection with WA1, confirming previous reports of an antiviral role of ORF8 in epithelial cell infection (*18*, *50*) (Fig. 3B). Consistent with these findings, plaque assays demonstrated that WA1-StopORF8 infection also produced higher titers of infectious viral titers than WA1 infection in AT2 monocultures (Fig. 3B). Finally, we infected co-cultures in which MDMs were grown on the bottom and AT2 cells on the upper chamber of the Transwell plate. In co-culture conditions with macrophages (Fig. 3C), AT2 cells exhibited a 20- (*PP* = 0.0468) and 7-fold (*PP* = 0.0246) increase in viral mRNA expression at 48- and 72-h postinfection, respectively, compared to AT2 cells cultured alone (Fig. 3B). Consistent with these findings, plaque assays demonstrated that WA1 infection under co-culture conditions produced the highest infectious viral titers among all experimental conditions tested (Fig. 3C). These results suggest that ORF8’s promotion of macrophage infection in allowing efficient viral replication in lung epithelial cells.

**Fig. 3.**
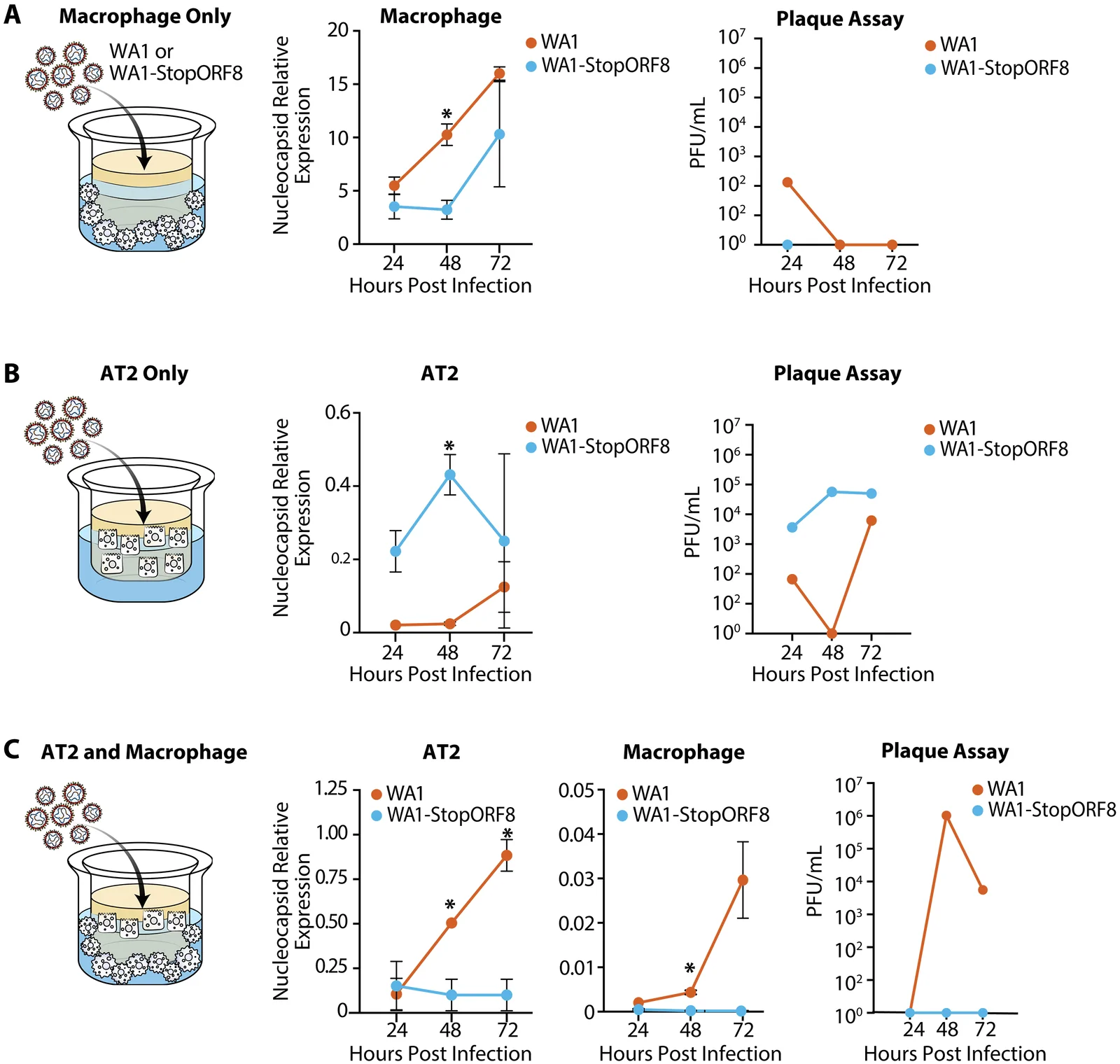
Viruses containing ORF8 enable SARS-CoV-2 to fully replicate in AT2 cells and macrophages. (**A**) MDMs were seeded on the bottom of 24-well Transwell plates and replication-competent SARS-CoV-2 WA1 or WA1-StopORF8 was added at MOI = 1. After 2 h, the cells were washed and fresh medium added. After 24, 48, and 72 h, the cells were collected and lysed. SARS-CoV-2 *N* mRNA in cell lysate was quantified by RT-qPCR normalized to HPRT. Plaque assays were performed using culture supernatants collected from each well of the WA-1 and WA-1-StopORF8 groups. Data are presented as mean values. (**B**) Primary human alveolar type 2 (AT2) cells were seeded in the upper chamber of 24-well Transwell plates. The two virus strains were added and analyzed as in (A). (**C**) MDMs and AT2 cells were co-cultured, with MDMs in the bottom and AT2 cells in the upper chamber of a 24-well Transwell plate. The two virus strains were added, washed after 2 h, and cells were analyzed separately by qPCR at 24, 48, and 72 h later. Data are presented as 2^-ΔCt^ (means ± SD of three independent experiments). Significance was assessed by two-tailed Student’s *t* test. *P < 0.05. The image was created in BioRender. Matsui, Y. (2026) https://biorender.com/jcwydga.

### Extracellular ORF8 shifts macrophages to an M2-like phenotype

IL-17 exerts dual functions, activating macrophages to protect the homeostatic state of tissues while also promoting inflammatory disease (*51*, *52*). We hypothesized that ORF8 reprograms macrophage phenotypes through mechanisms extending beyond modulation of ACE2 expression. To assess whether ORF8 affects macrophage phenotypes, we exposed unstimulated macrophages (referred to as M0), classically activated macrophages (M1), and alternatively activated macrophages (M2) to extracellular ORF8 and performed proteomic profiling at 12 and 48 h, quantifying 5723 total proteins in at least one condition (Fig. 4A, table S1). M0 macrophages showed increased CD206, a representative marker of M2 macrophages, at 48 h after ORF8 exposure and reduced DDX60 and IRF3, components of the RIG-I–dependent type-I interferon pathway (Fig. 4B, left panel). In M1 macrophages, CCR7, a representative marker of M1 macrophages, was downregulated, whereas HMOX1, MMP9, CHI3L1, and FGL2, proteins enriched in M2 macrophages, were upregulated (Fig. 4B, middle panel). In M2 macrophages, ORF8 preferentially amplified an M2-leaning profile, increasing mitochondrial/OXPHOS proteins (MRPL23, TIMM8A, COX5B, CYCS), antioxidant/stress-response factors (PRDX1, HPX, AK4), and immunoregulatory components (CSF1, LGALS3, CASP9, UBE2N), together with lipid/metabolic–stress proteins (APOC3, SRI), without broad induction of inflammatory markers (Fig. 4B, right panel). Across activation states, nine proteins—MMP9, APOC3, ITIH4, PVR, CASP9, CSF1, CYP1B1, MRPL23, and RUFY2—were upregulated in at least two macrophage states, mapping to pathways of tissue repair/remodeling, lipid/xenobiotic metabolism, mitochondrial function, immune regulation, cell-death signaling, and vesicular trafficking (Fig. 4, B and C). Collectively, these data indicate a shift toward an M2-like profile enriched for tissue-repair and lipid-metabolism pathways, with inflammatory signatures attenuated. Gene Ontology (GO) analysis indicated an enrichment in multiple immune-response pathways for M1 macrophages at 48 h post extracellular ORF8 exposure (fig. S3, table S2), therefore we analyzed polarity-associated mRNA changes in M1 cells using reverse transcription quantitative PCR (RT-qPCR). Exposure to extracellular ORF8 increased mRNA levels of M2-associated genes—*ARG1*, *VEGFA*, *IL10*, and *MRC1* (CD206)—whereas transcripts of interferon-pathway genes *IRF7*, *OAS1*, and *CXCL10* were reduced (Fig. 4D). For functional assays, phagocytosis was evaluated using pHrodo *E. coli* bioparticles. M1 macrophages pre-exposed to ORF8 exhibited increased fluorescence relative to controls, consistent with greater phagosome acidification/uptake (Fig. 4E). Furthermore, with lipopolysaccharide (LPS) at 1 ng/mL, IL-10 secretion by ORF8-exposed M1 macrophages did not differ significantly from unexposed controls; at 10 and 100 ng/mL it increased 9.2-fold (*PP* = 0.0005) and 3.6-fold (*PP* = 0.0026), respectively, as quantified using an enzyme-linked immunosorbent assay (ELISA) of culture supernatants (Fig. 4F).

**Fig. 4.**
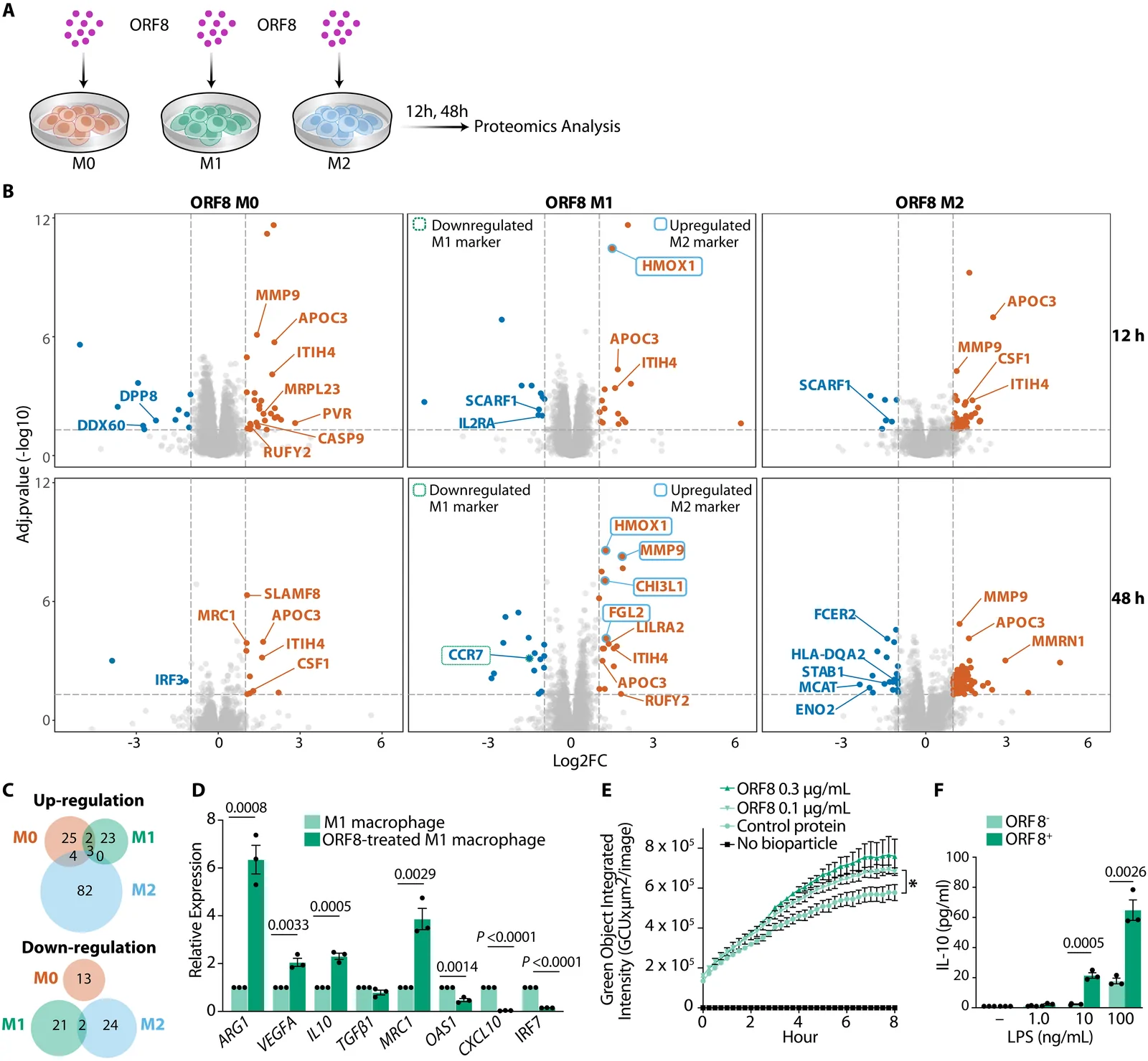
Extracellular ORF8 shifts macrophages to an M2-like phenotype. (**A**) Schematic of the proteomic analysis performed on MDMs (M0, M1, M2) cultured in the presence of extracellular ORF8. Created in BioRender. Matsui, Y. (2026) https://biorender.com/dr6wz9c (**B**) Volcano plots depicting differential protein expression in macrophages of three polarization states (M0, M1, M2) at 12- and 48-h following exposure to ORF8. The *x*-axis represents log_2_ fold change (log_2_FC), and the *y*-axis represents adjusted *P* values. Proteins to the right (red) are significantly upregulated, whereas those to the left (blue) are significantly downregulated in response to ORF8. Significance thresholds were defined as adjusted *P* < 0.05 and |log_2_FC| > 1. (**C**) Venn diagrams showing proteins significantly upregulated (upper) or downregulated (lower) in response to ORF8 exposure across three macrophage subtypes. (**D**) MDMs (M1) were incubated with ORF8 (1 μg/mL) or vehicle for 48 h. Expression of the indicated genes was quantified by RT-qPCR normalized to HPRT. (**E**) MDMs (M1) were cultured at 37°C for 8 h in the presence of extracellular ORF8 or SARS-CoV-2 N protein (control), together with pHrodo *E. coli* bioparticles. Fluorescence was monitored and quantified using the Incucyte live-cell analysis system. *P < 0.05. (**F**) MDMs (M1) was incubated with ORF8 or vehicle for 48 h, then LPS (1, 10, or 100 ng/mL) was added to the cell culture medium. The cell supernatant was harvested 24 h later and tested with IL-10 ELISA assay. Data are presented as fold change relative to unstimulated ± SD of three independent experiments. Significance was assessed by two-tailed Student’s *t* test.

### IL17RA antibody limits viral replication and lung pathology in ORF8-SARS-CoV-2-infection mice

To determine whether ORF8 influences viral replication and ACE2 expression in lung cells under in vivo conditions, C57BL/6 wild-type mice were infected with mouse-adapted SARS-CoV-2 WA1 (ORF8+), the StopORF8 WA strain, or the StopORF8 together with intraperitoneally administered recombinant ORF8 (ORF8 IP). Two days after infection, lungs were harvested, and macrophages and epithelial cells were isolated by antibody-based selection (Fig. 5, A and B). mRNA levels of the viral *N* gene and *Ace2* were quantified by RT-qPCR. In macrophages, *N* gene expression was increased 5.0-fold in WA1-infected mice (*P* = 0.0428) and 5.2-fold in ORF8 IP mice (*P* = 0.0339) compared with StopORF8-infected mice (Fig. 5B). *Ace2* expression was also elevated 4.3-fold in ORF8 IP mice (*P* = 0.0327), and WA1-infected mice showed a similar upward trend (Fig. 5C). In epithelial cells, *N* gene expression was 10-fold higher in WA1-infected mice (*P* = 0.0038) and 5.6-fold higher in ORF8 IP mice (*P* = 0.0474) compared with StopORF8-infected mice (Fig. 5E), whereas *Ace2* expression showed no significant differences between groups (Fig. 5F). To evaluate the therapeutic potential of IL-17RA blockade, we repeated these experiments on an additional cohort in which IL17RA antibody or a control IgG was administered intraperitoneally (Fig. 5G). In this cohort, infectious viral titers were 5.1-fold higher in the WA1 group (*P* = 0.0008) and 5.8-fold higher in the ORF8 IP group (*P* < 0.001) compared with the StopORF8 group in the absence of brodulamab. IL17RA antibody treatment reduced viral titers by 3.4-fold in the WA1 group (*P* = 0.0035), eliminating the difference from the StopORF8 group, whereas the StopORF8 group itself showed no response to IL17RA antibody. In contrast, the ORF8 IP group exhibited a 7.3-fold reduction in viral titer upon treatment with IL17RA antibody (*P* < 0.0001) (Fig. 5H). Histological analysis of lung tissue by haematoxylin–eosin staining and caspase-1 immunohistochemistry (IHC) revealed reduced air space and increased numbers of caspase-1–positive cells, including those in the airways, in the WA1 and ORF8 IP groups compated to the StopORF8 group (Fig. 5I), but these differences were abolished after intraperitoneal IL17RA antibody treatment (Fig. 5J). The area of caspase-1–positive regions was also elevated in WA1 and ORF8 IP groups and decreased following IL17RA antibody (Fig. 5K). In addition, Trichrome staining of WA1-infected lungs showed reduced fibrotic areas after IL17RA antibody treatment on five days after infection (Fig. 5, L and M).

**Fig. 5.**
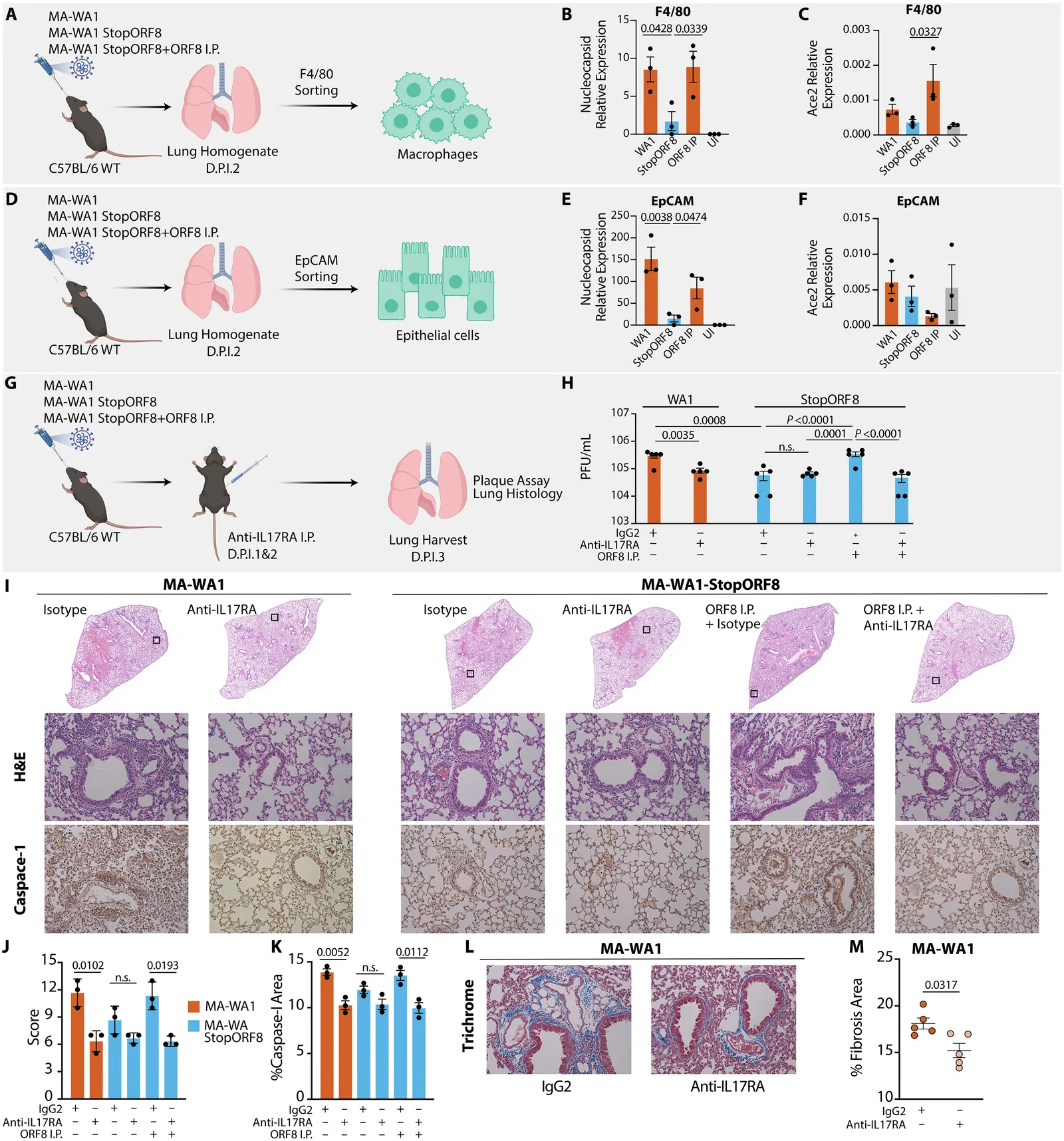
IL17RA Antibody Limits Viral Replication and Lung Pathology in ORF8-positive SARS-CoV-2 Infection. (**A** to **F**) C57BL/6 wild-type mice were infected with mouse-adapted (MA) WA1 (10^4^ PFU), MA-WA1-StopORF8, or WA1-StopORF8 supplemented with 10 μg of extracellular ORF8 administered intraperitoneally (IP) at 1-day postinfection (DPI 1). At 2 DPI, F4/80^+^ macrophages (A) and EpCAM^+^ epithelial cells (D) were isolated by positive selection, and the expression of SARS-CoV-2 *N* [(B) and (E)] and *Ace2* [(C) to (F)] mRNA was quantified by RT–qPCR. (**G** and **H**) C57BL/6 J mice were infected with mouse-adapted WA1 (10^4^ PFU), WA1-StopORF8, or WA1-StopORF8 supplemented with IP injection of 10 μg extracellular ORF8 at 1 and 2 DPI. Each group was further divided into two subgroups, receiving either IL17RA antibody (100 μg, IP at DPI 1 and 2) or IgG1 as an isotype control. In the ORF8-treated group, IL17RA antibody was administered IP 2 h after ORF8 injection. At 3 DPI, the lung tissue was homogenized, and infectious viral titers were quantified by plaque assay. Significance was assessed by one-way ANOVA with Kruskal-Wallis test with Dunn’s multiple comparison tests. (**I**, **J** and **K**) Lungs collected using the same procedure were fixed, stained with hematoxylin and eosin (H&E) and caspase-1 IHC, and caspase-1–positive areas were quantified by image analysis. (**L** and **M**) In MA-WA1–infected mice, the extent of fibrosis was assessed by Masson’s trichrome staining at 5 DPI. Data are presented as mean ± SD (*n* = 5 mice per group). Significance was assessed by two-tailed Student’s *t* test. *P < 0.05. (A), (D), and (G) created in BioRender. Matsui, Y. (2026) https://biorender.com/4owlfqk.

In an independent infection experiment using aged BALB/c mice, which are susceptible to mouse-adapted SARS-CoV-2 infection (*53*), mice infected with MA-WA1-StopORF8 exhibited only mild weight loss. In contrast, MA-WA1-infected mice developed more severe disease, with 2 of 5 animals exceeding the predefined euthanasia threshold of 15% body weight loss (fig. S4A). Consistent with these clinical findings, lung plaque assays revealed significantly higher infectious viral titers in MA-WA1-infected mice compared with MA-WA1-StopORF8-infected mice (fig. S4B). Based on these results, we propose a working model in which ORF8 engages IL-17RA signaling in macrophages to promote viral replication and lung pathology (Fig. 6).

**Fig. 6.**
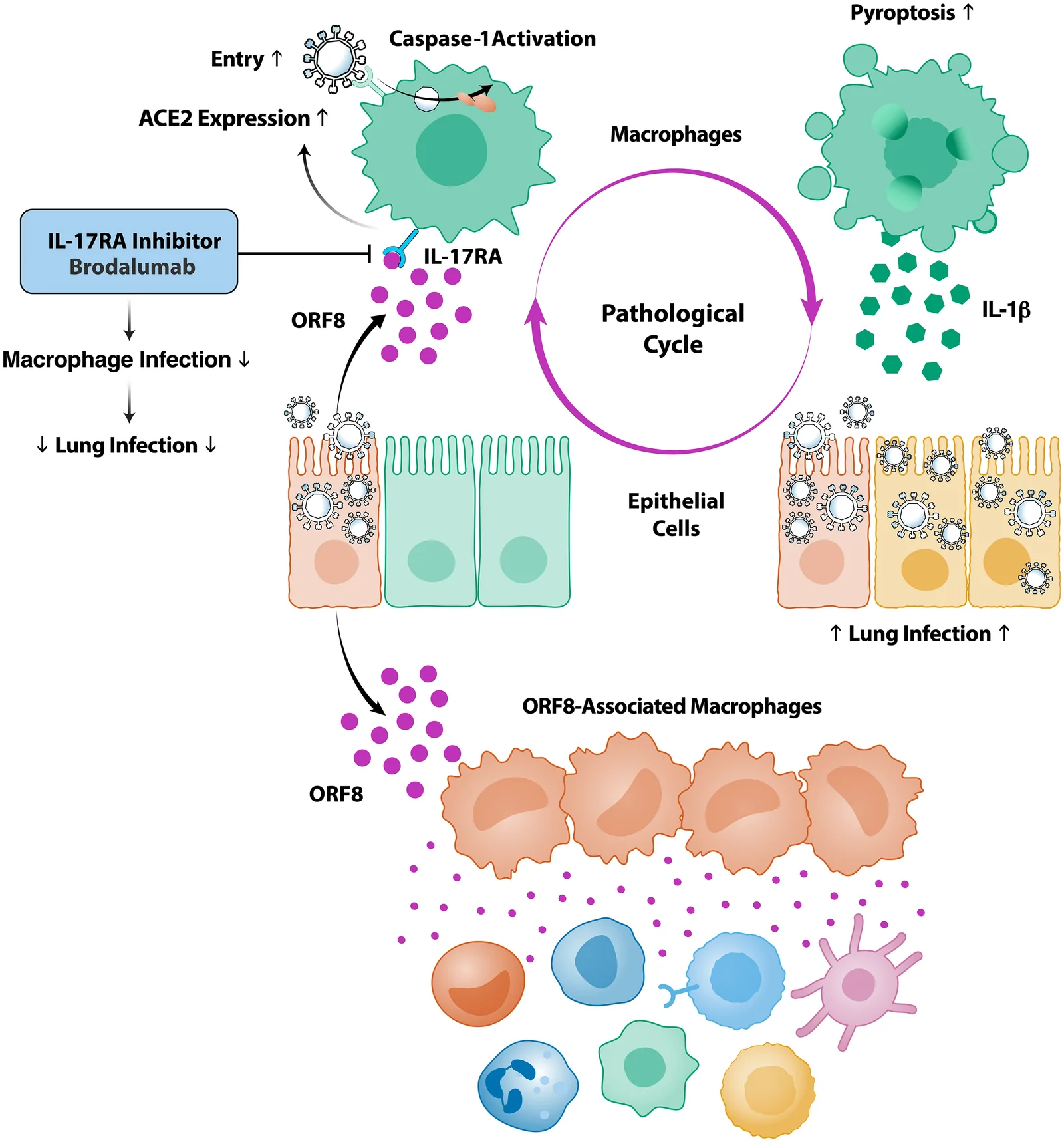
Model of ORF8-driven macrophage reprogramming enhancing SARS-CoV-2 epithelial infection. Secreted ORF8 engages IL-17RA on macrophages, upregulating ACE2 and enhancing viral uptake. Infection triggers caspase-1 activation and pyroptosis, releasing IL-1β that increases epithelial susceptibility to SARS-CoV-2. ORF8 also reprograms macrophages toward an immunosuppressive phenotype. Together, these events create a vicious cycle that amplifies viral replication and disease severity. IL-17RA blockade disrupts this loop and mitigates pneumonia.

## DISCUSSION

Our study identifies macrophages as key cellular targets of SARS-CoV-2 ORF8 and uncovers mechanisms by which ORF8 promotes viral spread while undermining host defense. We show that secreted ORF8 upregulates ACE2 in macrophages, markedly increasing their susceptibility to infection and pyroptotic cell death. This creates a feedforward loop in which infected macrophages increase epithelial permissiveness to infection, amplifying viral dissemination within the respiratory tract. Mechanistically, our data indicate that ORF8 induces ACE2 expression through an IL-17RA–TRAF6–JNK signaling axis associated with sustained AP-1 activation. Transcriptomic analyses revealed rapid induction of inflammatory and IL-17-associated gene programs following ORF8 stimulation, while pharmacological inhibition of IL-17RA, TRAF6, or JNK strongly suppressed ORF8-induced ACE2 expression. Notably, our reporter assays demonstrated that ORF8-induced NF-κB activation was rapid but transient, declining to near-baseline levels within 24 h, whereas AP-1 activation progressively increased over time. The rapid attenuation of NF-κB signaling, together with induction of multiple negative-feedback regulators suggests that ORF8-triggered NF-κB activation is tightly self-limited following the initial inflammatory response. In contrast, sustained AP-1 signaling may play a dominant role in maintaining ACE2 transcriptional upregulation. Previous studies have reported that macrophages express minimal or undetectable levels of IL-17RC (*54*), raising the possibility that ORF8 may engage IL-17RA signaling through a noncanonical mechanism. In addition, previous reports have suggested that only non-glycosylated ORF8 is capable of binding IL-17RA (*55*). Because recombinant ORF8 produced in mammalian expression systems likely contains heterogeneous glycosylation states, differences in ORF8 production methods may substantially influence downstream macrophage responses. Indeed, some groups have reported minimal transcriptional responses following ORF8 stimulation of macrophages (*56*). Such discrepancies may reflect differences in ORF8 synthesis and purification methods, macrophage differentiation and culture conditions, or the timing of sample collection following stimulation. How ORF8 functionally interacts with the IL-17 receptor complex in macrophages remains an important subject for future investigation. Consistent with this model, our in vivo experiments demonstrated that blockade of IL-17RA significantly reduced infectious viral titers to levels comparable to those observed with StopORF8 infection and improved disease outcomes.

At the same time, ORF8 reprograms macrophages from an inflammatory M1 profile toward an immunosuppressive M2-like state. Macrophages characterized by high CD206 expression, a hallmark of the M2-like state, are thought to play a role in SARS-CoV-2 pathogenesis (*21*, *29*, *37*). This shift, reinforced by IL-10, which is known to upregulate ACE2 on macrophages (*42*), mirrors the behavior of tumor-associated macrophages in suppressing local immune responses. Although pyroptotic inflammation and M2-like polarization appear contradictory, together they establish an immunopathological environment that favors viral replication while blunting antiviral immunity. Importantly, pyroptosis is accompanied by dysregulated release of IL-1β, which is known to drive aberrant AT2 self-renewal and promote fibrotic remodeling (*48*, *57*). This association raises the possibility that ORF8 contributes to post-acute sequelae of COVID-19 (PASC) by allowing macrophages to sustain inflammation (*58*) beyond the acute phase.

Finally, our co-culture experiments highlight the importance of epithelial–macrophage cross-talk in disease progression. ORF8 not only enhances pyroptosis and viral uptake in macrophages but also increases epithelial permissiveness through macrophage-derived signals. In agreement with our findings, previous work demonstrated synergistic cytokine induction in macrophage–lung epithelial cell co-culture systems in the presence of ORF8 (*55*). These observations are consistent with our proposed pathological cycle in which ORF8-mediated ACE2 upregulation enhances macrophage infection, leading to excessive inflammatory cytokine production that further promotes epithelial cell susceptibility and viral dissemination. Interestingly, the marked discrepancy between intracellular nucleocapsid mRNA levels and the infectious viral titers recovered from macrophage monocultures (Fig. 3A) suggests that only a limited subset of macrophages that internalize viral particles supports productive viral replication. In this context, ORF8-mediated enhancement of viral uptake through ACE2 upregulation may critically increase the likelihood of establishing replication-competent infection in macrophages. Our findings may help reconcile previous conflicting reports regarding macrophage infection by SARS-CoV-2. Together, these findings identify ORF8 as a central driver of epithelial–immune cell interactions in COVID-19, positioning it as both a mechanistic link between viral replication and immune evasion, and a promising therapeutic target.

Several limitations of this study should also be acknowledged. Although macrophage infection was confirmed by immunostaining analyses (fig. S1D), the overall frequency of infected macrophages remained relatively low and varied substantially among donors. Such variability may reflect differences in donor-specific innate immune states or prior immunological exposures. In particular, the potential contribution of trained innate immune memory to macrophage susceptibility and inflammatory responses following SARS-CoV-2 exposure warrants further investigation. Because ORF8-enhanced infection rapidly induced pyroptotic cell death, accurate quantification of infected macrophages over time was technically challenging. The rapid loss of infected cells may therefore lead to underestimation of the true extent of macrophage infection and viral replication dynamics.

## MATERIALS AND METHODS

### Ethics statement

All research conducted in this study complies with all relevant ethical regulations. All experiments conducted with replication-competent viruses were performed in a certified biosafety level 3 (BSL3) laboratory and experiments were approved by the Institutional Biosafety Committee of the University of California, San Francisco and the Gladstone Institutes. All protocols concerning animal use were approved (AN203103–00) by the Institutional Animal Care and Use Committees of the University of California, San Francisco and the Gladstone Institutes and conducted in strict accordance with the National Institutes of Health Guide for the Care and Use of Laboratory Animals. All human samples utilized for this project were de-identified, not used to conduct human subject research, and were therefore IRB exempt.

### ORF8 production

The ORF8-Flag expression construct was generated by ChemPartner. Recombinant C-terminal Flag-tagged ORF8 protein was expressed in HEK293 cells in a 2-L suspension culture for 7 days and purified using anti-Flag affinity purification followed by Superdex 200 size-exclusion chromatography. The purified protein was formulated in 50 mM Tris, 200 mM NaCl, and 1 mM DTT (pH 8.0). Protein purity was estimated to be approximately 90% by SDS–PAGE analysis.

The amino acid sequence of the recombinant ORF8-Flag protein was as follows: MKFLVFLGIITTVAAFHQECSLQSCTQHQPYVVDDPCPIHFYSKWYIRVGARKSAPLIELCVDEAGSKSPIQYIDIGNYTVSCSPFTINCQEPKLGSLVVRCSFYEDFLEYHDVRVVLDFIAIAGAAADYKDHDGDYKDHDIDYKDDDDK.

### SARS-CoV-2 propagation and infection

The SARS-CoV-2 isolate USA-WA1/2020 was produced by cloning into a molecular clone expression plasmid, followed by transfection into BHK-21 cells (ATCC). The WA1-stopORF8 clone was generated by site-directed mutagenesis as described previously (*39*). These viruses were then cultured in Vero E6 cells (ATCC) capable of expressing human ACE2 and TMPRSS2 receptors on the cell membrane. The cells were supplemented with a medium containing 2% FBS, D-glucose (4.5 g/L), 4 mM L-glutamine, 10 mM nonessential amino acids, 1 mM sodium pyruvate, and 10 mM HEPES. This process involved creating two successive virus stocks. Three days postinfection, the supernatant, which contained the propagated virus, was filtered through an Amicon Ultra 15 (100-kDa) centrifugal filter (Millipore Sigma) at approximately 4,000 rpm for 20 minutes. The flow-through was discarded, and the virus was resuspended in DMEM. The infectivity titer of SARS-CoV-2 was determined using a plaque assay on Vero E6-ACE2-TMPRSS2 cells in minimal essential medium enhanced with 2% FBS, 4 mM L-glutamine, 0.2% bovine serum albumin, 10 mM HEPES, 0.12% NaHCO_3_, and 0.7% agar. The multiplicity of infection (MOI) values used in the experiments were based on titers obtained from these plaque assays. All procedures involving live SARS-CoV-2 were conducted in the BSL-3 facility at Gladstone Institutes. This facility is certified by both the CDC and USDA and conforms to the institutional biosafety requirements. For ACE2-targeted infection-inhibition assays, cells were preincubated with an anti-human ACE2 antibody (R&D Systems) or an-isotype-matched human IgG2 control (Caltag Laboratories). For macrophage assays targeting IL-17RA, cells were preincubated with brodalumab (Selleckchem) or an isotype-matched human IgG2 control (Caltag Laboratories).

### Plaque assay

Supernatants from cell cultures were assessed for the formation of viral particles in both cell and in organoid experiments. In summary, Vero-TMPRSS2 cells were seeded and allowed to incubate overnight. Subsequently, cells were exposed to 10^−1^ to 10^−6^ dilutions of the respective homogenates or supernatants in serum-free DMEM. Following a 1-hour absorption period, a 2.5% Avicel (RC-591, Dupont) overlay was applied to the wells. After 72 h, cells were fixed with 10% formalin for 50 min and stained with crystal violet. Plaques were counted, and infectious viral titers were calculated and expressed as plaque-forming units (PFU)/mL. Data analysis was carried out using GraphPad Prism version 10.2.0.

### Production of virus-like particles and assessment of transduction efficiency by luciferase assay

Plasmids CoV2-N (10 μg), CoV2-M-IRES-E (5 μg), CoV-2 Spike (24 ng), and Luc-T20 (15 μg) were mixed in a total volume of 2 mL of Opti-MEM (Gibco) (*38*). To this DNA solution, 90 μg of PEI was added, resulting in a final volume of 2 mL in Opti-MEM. The transfection mixture was then allowed to incubate at room temperature for 20 minutes. Following this incubation, the mixture was applied to 293 T cells (ATCC) (1.2 × 10^7^) in T175 flasks containing DMEM. The medium in these flasks was replaced 24 h post-transfection. At 48 h after transfection, the supernatant, which contained the virus-like particles (VLPs), was harvested, and subsequently passed through a 0.45 μm syringe filter to ensure purity. For the luciferase assay, 100 μl of this filtered supernatant was dispensed into each well of a 96-well plate, which already contained 30,000 MDMs per well. These cells were left to adhere overnight, allowing for the incorporation of the VLPs. The following day, the supernatant was removed, and the cells were washed with PBS (Corning). Then, 20 μL of cell lysis buffer (Promega) was added to each well, and the plate was gently rocked at room temperature for 15 minutes to facilitate cell lysis. The resulting lysate was then transferred to a white 96-well plate. To each well, 50 μL of reconstituted luciferase assay buffer was added and mixed thoroughly with the lysate. The luminescence generated by this reaction was measured immediately using EnSpire plate reader (PerkinElmer) to assess the efficiency of transfection and VLP incorporation.

Production of VSVΔG (G protein-deficient vesicular stomatitis virus) SARS-CoV-2 Spike pseudotyped virions and assessment of transduction efficiency by Renilla luciferase assay.

For the preparation of virions, 293 T cells were transfected with the spike plasmid, followed by inoculation with a generated working stock of VSVΔG-rLuc*G containing an integrated Renilla luciferase reporter gene to generate the pseudotyped VSVΔG-rLuc*SARS-CoV-2 (*59*). Pseudotyped virions were generated using Spike plasmids harboring mutations found in the WT SARS-CoV-2 Spike (Wuhan-Hu-1). For the Renilla luciferase assay, 10 μL of the virion stock was dispensed into each well of a 24-well plate, which already contained 10^5^ Calu-3 cells per well. These cells were left to adhere overnight, allowing for the incorporation of the virions. The following day, the supernatant was removed, and the cells were washed with PBS (Corning). Then, 100 μL of cell lysis buffer (Promega) was added to each well, and the plate was gently rocked at room temperature for 15 minutes to facilitate cell lysis. The resulting lysate was then transferred to a white 96-well plate. To each well, 50 μL of reconstituted luciferase assay buffer was added and mixed thoroughly with the lysate. The luminescence generated by this reaction was measured immediately using EnSpire plate reader (PerkinElmer) to assess the efficiency of transduction and the pseudovirus incorporation.

### Production of SARS-CoV-2 replicon particles

The pBAC SARS-CoV-2 Δ Spike WT plasmid (1 μg), was transfected into BHK-21 cells along with N and S expression vectors (0.5 μg each) in 24-well. The supernatant was replaced with fresh growth medium 16 hours post-transfection. The supernatant containing single-round infectious particles was collected and 0.45 μm-filtered 72 hours post-transfection. The supernatant was subsequently used to infect macrophages (in 96-well plate). To measure luciferase activity, an equal volume of supernatant from infected cells was mixed with Nano-Glo luciferase assay buffer and substrate and analyzed on an Infinite M Plex plate reader (Tecan).

### Isolation and culture of human AT2 cells

Human lungs from brain-dead donors who were declined for transplantation were obtained through Donor Network West for research and transported to UCSF under cold storage conditions. Human alveolar type II (AT2) cells were isolated from donor lungs as previously described (*60*, *61*). Briefly, an uninjured lung lobe was selected based on gross inspection and chest imaging. The pulmonary vasculature was flushed, and distal airspaces were lavaged. Lung tissue was digested with elastase (13 U/mL, Worthington Biochemical Corp.), minced, filtered, and subjected to density gradient centrifugation. CD14^+^ cells were depleted, and AT2 cells were further enriched and purified by IgG panning.

Freshly isolated AT2 cells were seeded onto collagen I–coated Transwell inserts (0.4-μm pore size; Costar, Corning) in a 1:1 mixture of DMEM-H21 and Ham’s F-12 supplemented with 10% fetal bovine serum (FBS) at a density of 1 × 10^6^ cells per well. Cells reached confluence within 48 h. Medium in the apical compartment was removed daily to promote the formation of a tight epithelial monolayer. An air–liquid interface (ALI) was established 96–120 h after seeding, as indicated by the absence of fluid leakage from the basolateral compartment to the apical compartment. For coculture experiments, AT2 monolayers were washed with serum-free medium to remove residual serum prior to downstream experiments. MDMs were then seeded into the basolateral compartment of the Transwell system beneath the AT2 monolayer.

### Lung epithelial cell line

Calu-6 epithelial cells (ATCC), genetically modified to stably express human angiotensin-converting enzyme 2 (hACE2; OriGene), were cultured in RPMI 1640 medium (Invitrogen), enriched with 10% fetal bovine serum (Gibco). For VLP transduction experiments, these cells were seeded onto 24-well plates at a density of 5 × 10^4^ cells per well.

Calu-3 cells were maintained in high-glucose DMEM (Gibco) supplemented with 20% fetal bovine serum, 1% non-essential amino acids, 2 mM L-glutamine, 1 mM sodium pyruvate, 100 U/mL penicillin–streptomycin, and 1.5 g/L NaHCO_3_. For air–liquid interface (ALI) differentiation, Calu-3 cells were seeded onto 6-well Transwell inserts at a density of 5 × 10^5^ cells per insert. Culture medium in both the apical and basolateral compartments was replaced every 2 days. On day 7 after seeding, medium was removed from the apical compartment to establish ALI conditions. Cells were maintained under ALI culture for an additional 11 days and used for experiments on day 18 after initial seeding.

### Primary human macrophages

Primary human macrophages were generated from highly enriched peripheral monocytes. To obtain these cells, peripheral blood mononuclear cells (PBMCs) were first isolated from the blood of healthy donors (Vitalant) using Lymphoprep density gradient medium (StemCell Technologies). Subsequently, CD14^+^ monocytes were selectively extracted from the PBMCs through negative selection, employing a combination of an antibody mixture designed for human monocyte enrichment and a magnetic column separation system (STEMCELL Technologies). Purified monocytes were cultured at a density of 1 × 10^6^ cells/mL in serum-free Macrophage medium (StemCell Technologies), supplemented with M-CSF at a concentration of 50 ng/mL (PeproTech) for six days. For M1 polarization, monocyte-derived macrophages were stimulated with LPS (10 ng/mL) and IFN-γ (50 ng/mL) (both from PeproTech). For M2 polarization, cells were treated with IL-4 (10 ng/mL, PeproTech) following an 8-day differentiation protocol in the macrophage medium (STEMCELL Technologies).

### Inducing polarization in the THP-1 cell line

The THP-1 cell line was maintained in RPMI 1640 medium (Invitrogen), supplemented with 10% fetal bovine serum (Gibco) and 2 mM L-glutamine (Invitrogen). To initiate differentiation into macrophage-like cells, the cells were treated with phorbol myristate acetate (PMA, Sigma-Aldrich) at a concentration of 150 nmol/L (PeproTech) for 24 h. Following this, the cells underwent multiple washes. To induce differentiation into the M1 phenotype, 20 ng/mL IFN-γ and 10 ng/mL LPS were added to the culture for 72 h. For M2 phenotype differentiation, the cells were treated with 20 ng/mL IL-4 and 20 ng/mL IL-13 for the same duration.

### Macrophage phenotyping

The surface expression of CD14 (APC-Cy7), CD80 (PE), and CD206 (FITC) molecules (BD Biosciences) on macrophages was quantified using standard flow cytometry employing a BD LSR Fortessa X-20 instrument, with subsequent analysis carried out using FlowJo software (BD Biosciences). All macrophages were treated with Human BD Fc Block reagent (BD Biosciences) for 15 min before staining. Specific antibodies targeting these molecules, along with their respective isotype-matched control antibodies, were sourced from Caltag Laboratories.

Transwell co-culture infection assay 3 × 10^5^ human monocyte-derived macrophages were seeded onto Transwell plates using Macrophage medium (StemCell Technologies) enriched with M-CSF (50 ng/μL). Concurrently, an equal number of AT2 cells (3 × 10^5^) were cultured on the upper chamber of T-24 Transwell plate inserts (Corning) using DMEM high glucose 50%, F-12 50% mix medium (UCSF Media Production). The SARS-CoV-2 strain USA-WA1/2020 was then introduced to the cells at an MOI of 2. After 2 h, the medium was replaced with fresh medium. At 24, 48, and 72 h postinfection, cell lysates were harvested using TRIzol (Invitrogen), and the culture supernatants were stored at −80°C for further analysis by plaque assay.

### Reverse transcription quantitative PCR (RT-qPCR)

Total cellular RNA was extracted utilizing TRIzol Reagent (Invitrogen) following the manufacturer’s instructions. qRT–PCR was performed using 1 μg of total RNA with Luna Probe One-Step RT-qPCR 4× Mix with UDG (New England Biolabs) and TaqMan Gene Expression Assays (ACE2 Hs01085333_m1 and HPRT Hs02800695_m1, Thermo Fisher Scientific) according to the manufacturers’ instructions. To validate low-abundance transcript detection, selected samples were additionally analyzed following pre-amplification using the TaqMan PreAmp Master Mix (Thermo Fisher Scientific). Quantitative PCR reactions were performed using a qTOWER3 84G Real-Time PCR Thermal Cycler (Analytik Jena). For ARG1, VEGFA, IL10, TGFβ1, MRC1 (CD206), OAS1, CXCL10, IRF7, TMPRSS2, NRP1, FCGR3A, CTSL, and TLR4 expression analyses (table S3), cDNA was synthesized from total RNA using the iScript Reverse Transcription Supermix (Bio-Rad) according to the manufacturer’s instructions. Quantitative PCR was subsequently performed using Maxima SYBR Green qPCR Master Mix (Thermo Fisher Scientific).

MDMs were seeded at 1 × 10^5^ cells per well in 96-well plates. Cells were stimulated with ORF8 (0.1 or 0.3 μg/mL) or SARS-CoV-2 nucleocapsid protein (0.3 μg/mL; GenScript) and incubated with pHrodo Green *E. coli* Bioparticles (100 μg/mL; Sartorius). Phagocytic activity was monitored in real time by quantifying the green fluorescence signal using an Incucyte SX5 live-cell analysis system (Sartorius).

### Il-10 ELISA

ORF8 (Chempartner), at a concentration of 1 μg/mL, was added to the medium of MDM (M1) macrophages. After 48 h, LPS (PeproTech) at concentrations of 1, 10, or 100 ng/mL was introduced. Following 24 h, the medium was collected. This medium was then applied to anti-human IL-10 pre-coated 96-well plates, and an ELISA was conducted as per the manufacturer’s instructions (BioGems). The IL-10 concentration was determined using the standard curve.

### FLICA assay

MDMs were cultured with VLPs alone or with VLPs supplemented with recombinant ORF8 (1 μg/mL). Activated caspase-1 was detected using either green fluorescent FAM-FLICA or far-red fluorescent FLICA 660 reagents (ImmunoChemistry Technologies) according to the manufacturer’s instructions. After 30 min of incubation, cells were washed according to the manufacturer’s protocol and stained with Zombie Violet viability dye (BioLegend). Samples were subsequently analyzed using a BD LSRFortessa X-20 flow cytometer (BD Biosciences).

### NF-κB-/AP-1-reporter assay

Reporter virus particles were generated using the pSIRV transfer vector (Addgene) in an MMLV-based packaging system together with a VSV-G envelope plasmid. Secreted NanoLuc luciferase expression was driven by NF-κB or AP-1 response elements upstream of a minimal promoter and used as a readout of NF-κB and AP-1 signaling, respectively. These retroviral vectors were transduced into MDMs, followed by stimulation with ORF8 and/or IL-17 4 days later. At the time of sample collection, culture supernatants were completely removed, cells were washed three times with PBS, and fresh medium was added prior to downstream analysis.

### Proteomics analysis

Abundance proteomics sample preparation. Each of the 36 frozen cell pellets (sample details in table S4) was resuspended in 100 μL of urea lysis buffer (8 M urea, 150 mM NaCl_2_, 100 mM Tris) on ice, sonicated for 30 seconds, prior to one round of freeze thaw. Protein concentration of the resulting lysates was determined by Bradford assay. For each lysate, 50 μg was reduced with 2 mM tris(2-carboxyethyl)phosphine (Sigma, C4706) for 30 minutes at room temperature and alkylated with iodoacetamide final concentration 10 mM for 30 minutes at room temperature in the dark. Iodoacetamide was quenched by addition of DTT at final concentration 10 mM and incubation in the dark at room temperature for 30 minutes. Buffer exchange and protein digestion were performed on a KingFisher Flex unit using a protein aggregation approach. In this digestion protocol the setup is as follows: plate #1 stores the 96-well comb; plate #2 contains the lysate/bead mixtures; plate #3–5 contain 95% acetonitrile wash solutions; plates #6–7 contain 70% ethanol wash solutions; and plate #8 contains the digestion elution buffer. Prior to starting the automated runs, the lysate/bead mixtures are prepared by mixing together 20 μL MagReSyn Amine beads (Resyn Biosciences) with 100 μL of lysate and 280 μL of 100% acetonitrile (to plate #2). Protein aggregation on beads was induced by 4 cycles of mixing (1 min), pause (5 min), and collection of the magnetic beads (24 min total time). Beads with bound protein were then transferred to plate #3 and released into 150 μL of 95% acetonitrile and washed by 5 cycles of mixing. Washing cycle is repeated two times in plates #4 and #5. Beads are then transferred to plate #6 and released into 150 μL of 70% ethanol and washed by 5 cycles of mixing. This cycle was repeated once more in plate #7. Finally, protein bound beads were released into plate #8 with freshly prepared digestion elution buffer (150 μL of 50 mM ammonium bicarbonate (pH 7.8) with 0.5 μg Lys-C and 0.5 μg trypsin). Plate #8 with the digesting proteins was then sealed, and proteolytic digestion proceeded overnight at 37°C with agitation at 800 rpm (Eppendorf ThermoMixer). The resulting peptides were then filtered by 0.45 μm membranes, acidified with 5 μL formic acid, and then dried by vacuum centrifugation.

Protein abundance mass spectrometry (MS) data acquisition and analysis. For MS acquisition, dried samples were resuspended in 25 μL of sample buffer (0.1% formic acid in water) and 1 μL of peptide was injected on a Orbitrap Exploris 480 mass spectrometer (Thermo Fisher Scientific) coupled to a Vanquish Neo liquid chromatography instrument (Thermo Fisher Scientific). Briefly, peptides were separated on a 150 μm x 15 cm PepSep column (Bruker, 1.5 μm beads) over a 90 min gradient at a flow rate of 600 nL/minute as described in table S5. Buffer A consisted of 0.1% formic acid (FA) in water, and buffer B was 0.1% FA in 80% acetonitrile. Spectra were continuously acquired in a data-dependent acquisition (DDA) mode. One full scan was acquired in the Orbitrap (350–1250 m/z at 120,000 resolution with a normalized AGC target of 300%) followed by as many MS/MS scans as could be acquired on the most abundant ions in 2 seconds in the Orbitrap (1500 resolution; Normalized collision energy type; HCD collision energy of 28%; normalized AGC target of 200%, maximum injection time set to Auto, and isolation window of 1.6 m/z). Singly and unassigned charge states were rejected, and dynamic exclusion was enabled after n = 1 time, with an exclusion duration of 20 seconds (tolerance of ±10 ppm). Detailed MS acquisition parameters are reported in table S6. Raw MS files were analyzed using Fragpipe (version 21.1). MS/MS spectra were searched against the human proteome (UniProt reviewed, downloaded 09 May 2024; concatenated with equal sized reversed database and common contaminants) using default parameters (table S7).

Peptide ion intensities from the output of Fragpipe (table S7) were summarized to protein intensities using the R Bioconductor package MSstats (version 4.8.3). The functions dataProcess and groupComparison were applied with default settings, except for setting “MBimpute = FALSE” in dataProcess. Proteins with an adjusted p-value ≤0.05 and an absolute log_2_ fold change >1 were considered significant. Significantly changed genes were tested for Gene Ontology (GO) term enrichment, including Biological Process (BP), Molecular Function (MF), and Cellular Component (CC), using the enricher function from the clusterProfiler package (version 4.8.1). GO annotations were retrieved using clusterProfiler:get_GO_data. Enriched GO terms with adjusted p-values <0.1 were considered significant. To remove redundancy among GO terms, we constructed a GO term tree based on pairwise distances defined as 1 - Jaccard similarity coefficient of shared genes. The tree was cut at a height of h = 0.99 to identify clusters of similar terms. Within each cluster, the representative term was selected by choosing the broadest significant GO term.

### Bulk RNA sequencing

Monocytes (1 × 10^6^ cells) were seeded in 6-well plates and differentiated into macrophages. Cells were stimulated with ORF8 (1 μg/mL) for 3 h prior to analysis. Cell lysates were then prepared in 150 μL of DNA/RNA Shield (Zymo Research). Bulk RNA sequencing was performed by Plasmidsaurus. RNA quality was assessed prior to library preparation, and sequencing libraries were generated according to the manufacturer’s standard protocols. Libraries were sequenced on an Illumina platform. Reads were aligned to the human reference genome (GRCh38), and differential expression analysis was performed using DESeq2 with an adjusted *P* value cutoff of 0.05.

### C57BL/6 and BALB/c wild-type mouse SARS-CoV-2 infection model

All protocols concerning animal use were approved (AN203103–00) by the Institutional Animal Care and Use committees at the University of California, San Francisco and Gladstone Institutes and conducted in strict accordance with the National Institutes of Health Guide for the Care and Use of Laboratory Animal. C57BL/6 J mice were obtained from The Jackson Laboratory, and BALB/c mice were obtained from Charles River Laboratories. Mice were housed in a temperature- and humidity-controlled specific pathogen-free facility under a 12 h light/dark cycle with ad libitum access to water and standard laboratory chow. We constructed a mouse-adapted (MA)-SARS-CoV-2 (Spike: Q498Y/P499T54) using pGLUE (*39*). MA-SARS-CoV-2 (1 × 10^3^ PFU in 40 μL) was administered intranasally under anesthesia to 6–8-week-old mice. For MA-SARS-CoV-2 StopORF8 experiments, purified ORF8 protein (10 μg; ChemPartner) was administered intraperitoneally at 24 and 48 h post-vaccination. In prevention assays, wild-type mice received intranasal delivery of the peptide (25 μg in 40 μL), followed 30 min later by intranasal inoculation with MA-SARS-CoV-2 (1 × 10^3^ PFU in 40 μL). Mice were euthanized 24 h after peptide treatment. For intervention studies, IL17RA antibody (100 μg; R&D Systems) was administered intraperitoneally 2 h after ORF8 injection, and mice were euthanized 72 h after viral challenge. Mouse IgG1 isotype control (R&D Systems) was used as a negative control. For assessment of lung fibrosis, mice were euthanized 5 days after infection and lungs were collected for analysis.

F4/80^+^ macrophages and EpCAM^+^ epithelial cells were isolated from lungs 2 days after viral inoculation. Excised lungs were minced with scissors and digested in RPMI containing DNase I (100 μg/mL; Roche) and Liberase TL (10 μg/mL; Sigma-Aldrich), followed by mechanical dissociation using a gentleMACS Octo Dissociator (Miltenyi Biotec). Cell suspensions were separated with Anti-F4/80 MicroBeads and CD326 (EpCAM) MicroBeads (Miltenyi Biotec) and subjected to RT–qPCR analysis.

### Histology

Mouse lung tissues were fixed in 4% PFA (Sigma Aldrich) for 24 hours, washed three times with PBS and stored in 70% ethanol. All the stainings were performed at Histo-Tec Laboratory, Inc. (Hayward, CA). Samples were processed, embedded in paraffin, and sectioned at 4 μm. The slides were dewaxed using xylene and alcohol-based dewaxing solutions. Epitope retrieval was performed by heat-induced epitope retrieval of the formalin-fixed, paraffin-embedded tissue using 10 mM citrate-based pH 6 solution for 20 mins at 95°C. The tissues were stained for H&E and Caspase-1 (Abcam) and dried, coverslipped, and visualized using Axioscan 7 (ZEISS) at 10X and 40X. For Masson’s trichrome staining, nuclei were stained with Weigert’s iron hematoxylin solution, followed by staining with Biebrich scarlet-acid fuchsin solution to visualize cytoplasm and muscle fibers. Sections were then treated with phosphomolybdic-phosphotungstic acid solution and subsequently stained with aniline blue to label collagen fibers. Finally, slides were differenciated in 1% acetic acid, dehydrated in graded ethanol, cleared in xylene, and mounted with a resinous medium. All slides were interpreted by a board-certified pathologist. Caspase-1-positive cells and pulmonary fibrotic area were quantified in Fiji/ImageJ.
