# Supplementary material for: Secreted ORF8 reprograms macrophages to enhance SARS-CoV-2 infection of lung epithelial cells
Source: Sci Adv. 2026 Sep 18;12(38):eaee5421. doi: 10.1126/sciadv.aee5421 (PMC13588166; doi:10.1126/sciadv.aee5421)
Supplement: Supplementary file 1 [file sciadv.aee5421_sm.pdf]

Science Advances
AAAS

Supplementary Materials for

Secreted ORF8 reprograms macrophages to enhance SARS-CoV-2 infection of lung epithelial cells

Yusuke Matsui *et al.*

Corresponding author: Melanie Ott, melanie.ott@gladstone.ucsf.edu

Sci. Adv. **12**, eaee5421 (2026)
DOI: 10.1126/sciadv.aee5421

The PDF file includes:

Figs. S1 to S5
Legends for tables S1 to S7

Other Supplementary Material for this manuscript includes the following:

Tables S1 to S7

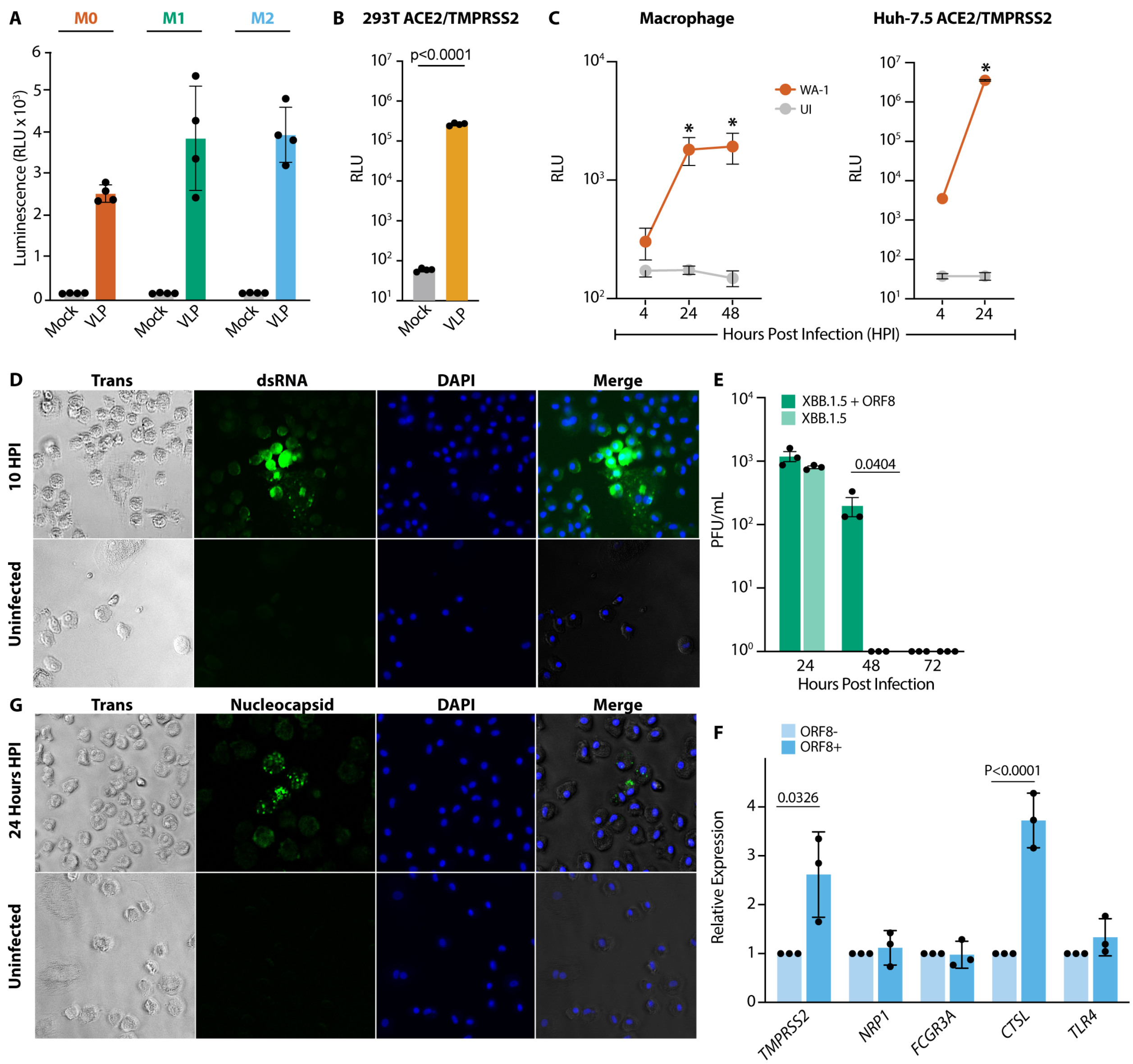

Figure S1. MDMs support limited and transient production of infectious SARS-CoV-2. (A) MDMs (M0, M1, and M2) were exposed to luciferase-encoding VLPs for 24 h, and luciferase activity was subsequently quantified. (B) 293T-ACE2/TMPRSS2 cells were exposed to luciferase-encoding VLPs for 24 h, followed by quantification of luciferase activity. (C) MDMs and Huh-7.5 ACE2/TMPRSS2 cells were exposed to replicons, and NanoLuc activity in the supernatant was quantified as a measure of SARS-CoV-2 RNA replication. *$p < 0.05$. (D) MDMs were infected with replication-competent SARS-CoV-2 at an MOI of 1; dsRNA was detected at 10 h (top), and N protein was detected at 24 h post-infection (bottom).

(E) MDMs were infected with SARS-CoV-2 XBB.1.5 or XBB.1.5 supplemented with extracellular ORF8 at an MOI of 2. Culture supernatants were collected at 24, 48, and 72 h post-

infection, and infectious viral titers were quantified by plaque assay and expressed as PFU/mL. Statistical significance was determined using two-tailed Welch's *t* test. (F) MDMs were treated with ORF8 (1 μg/mL). Expression of the indicated genes was quantified by qPCR normalized to HPRT. Data are presented as mean ± SD. n = 3 unless otherwise indicated; n = 4 for A and B. Statistical significance was determined using a two-tailed Welch's *t* test.

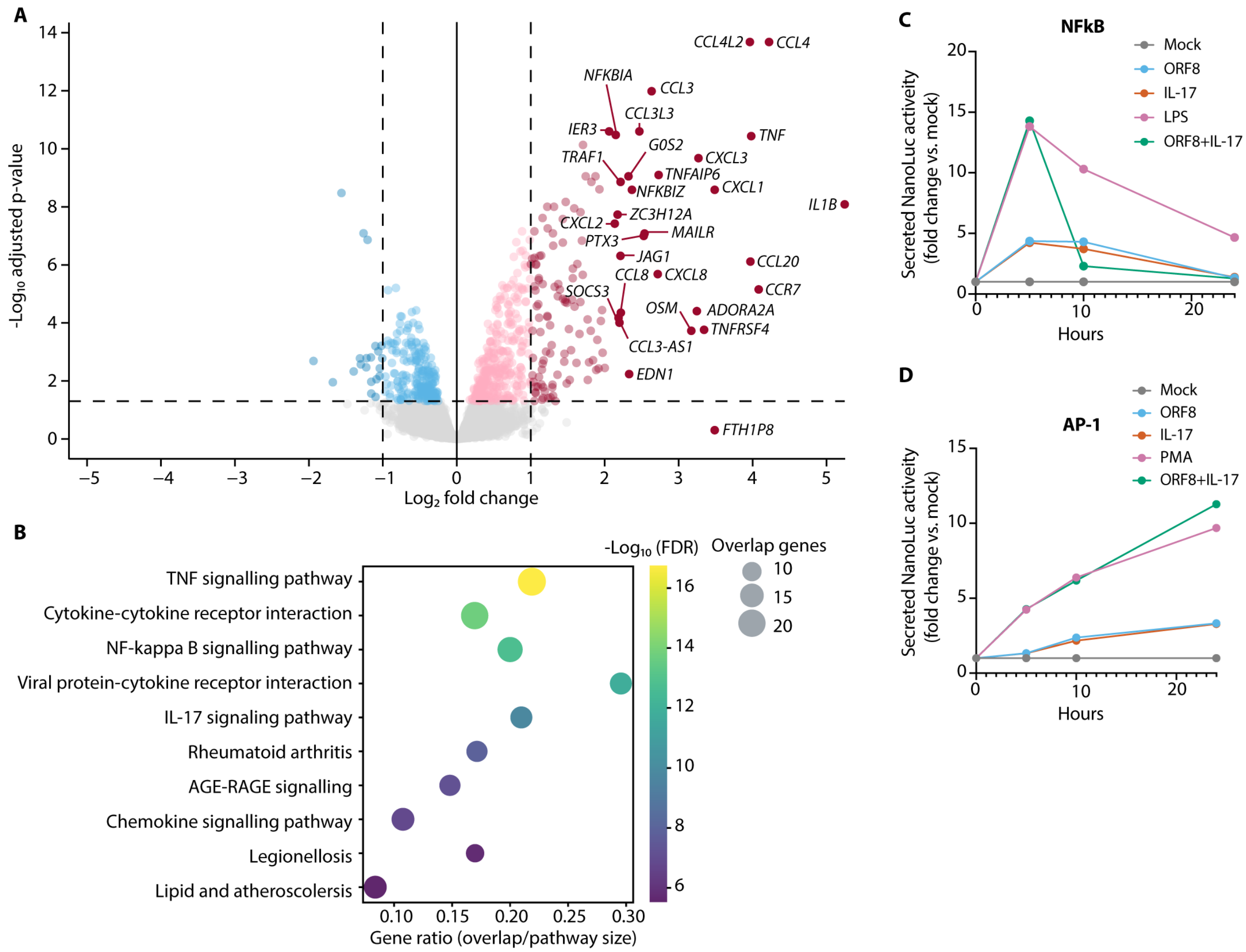

Figure S2. ORF8-induced NF-κB/AP-1 signaling responses in MDMs.

(A) MDMs were stimulated with ORF8 (1 μg/mL) for 3 h, followed by RNA-seq analysis in biological triplicates and comparison with mock-treated controls. Red dots indicate upregulated genes, blue dots indicate downregulated genes, and green dots indicate the top 30 upregulated genes. **(B)** KEGG pathway enrichment analysis of significantly upregulated genes (adjusted $P <$ 0.05, log2 fold change > 1) is shown as a dot plot. Dot size represents gene ratio, and color indicates false discovery rate (FDR). **(C and D)** MDMs transduced with NF-κB (C) or AP-1 (D) NanoLuc reporter retroviral particles were treated with ORF8 (1 μg/mL), IL-17 (200 ng/mL), LPS (100 ng/mL), or ORF8 plus IL-17. Culture supernatants were collected at 5, 10, and 24 h post-treatment, and NanoLuc activity was quantified. At the 5- and 10-h time points, supernatants were completely removed, cells were washed with PBS, and fresh medium containing the indicated stimuli was added. Experiments were performed in biological triplicates, and data are presented as mean values.

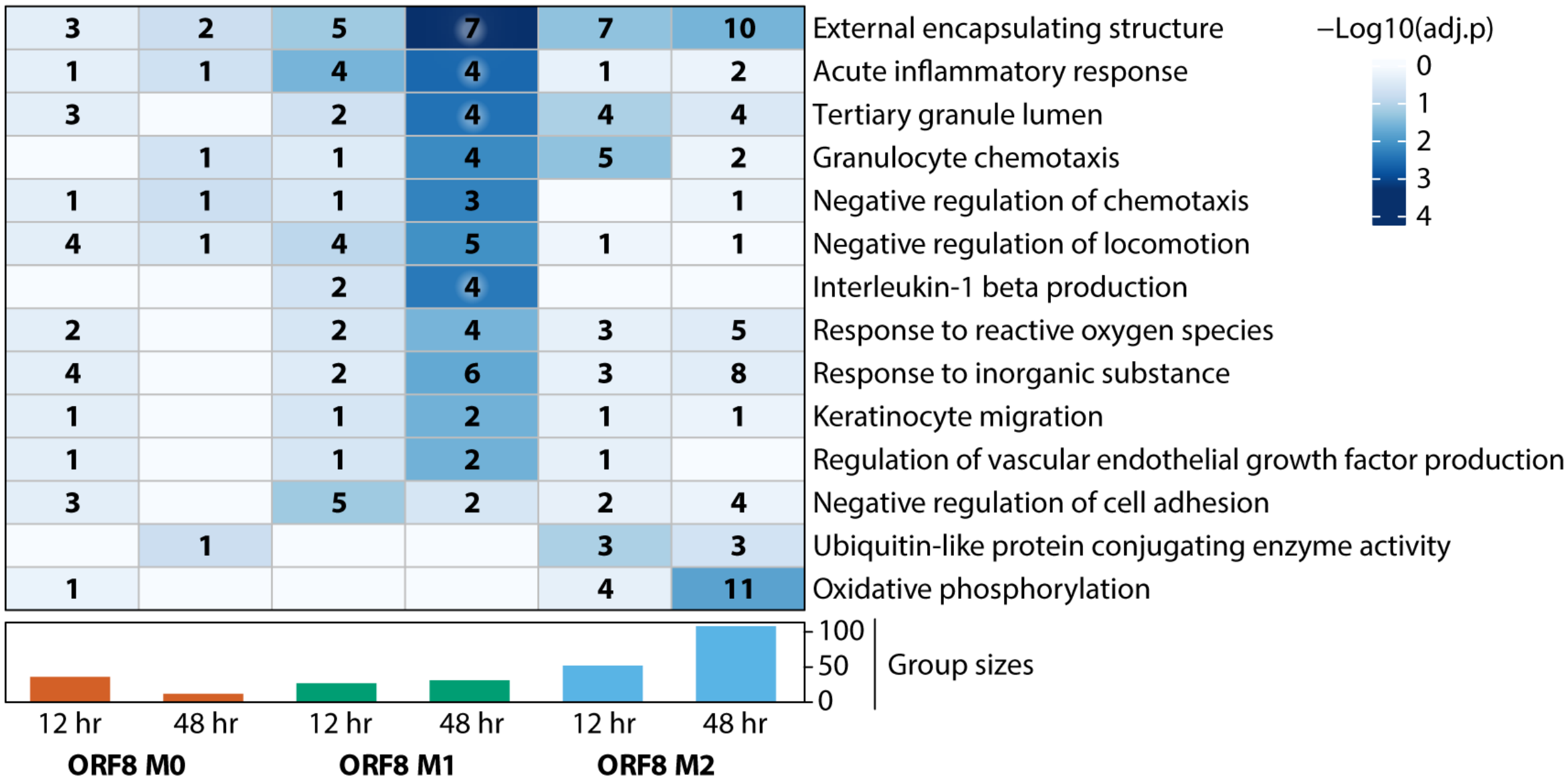

Figure S3. GO enrichment analysis of ORF8-exposed macrophages. Gene Ontology (GO) term enrichment analysis was performed on differentially expressed proteins identified by proteomic profiling of M0, M1, and M2 macrophages following exposure to extracellular ORF8. Significantly enriched biological processes, molecular functions, and cellular components are shown.

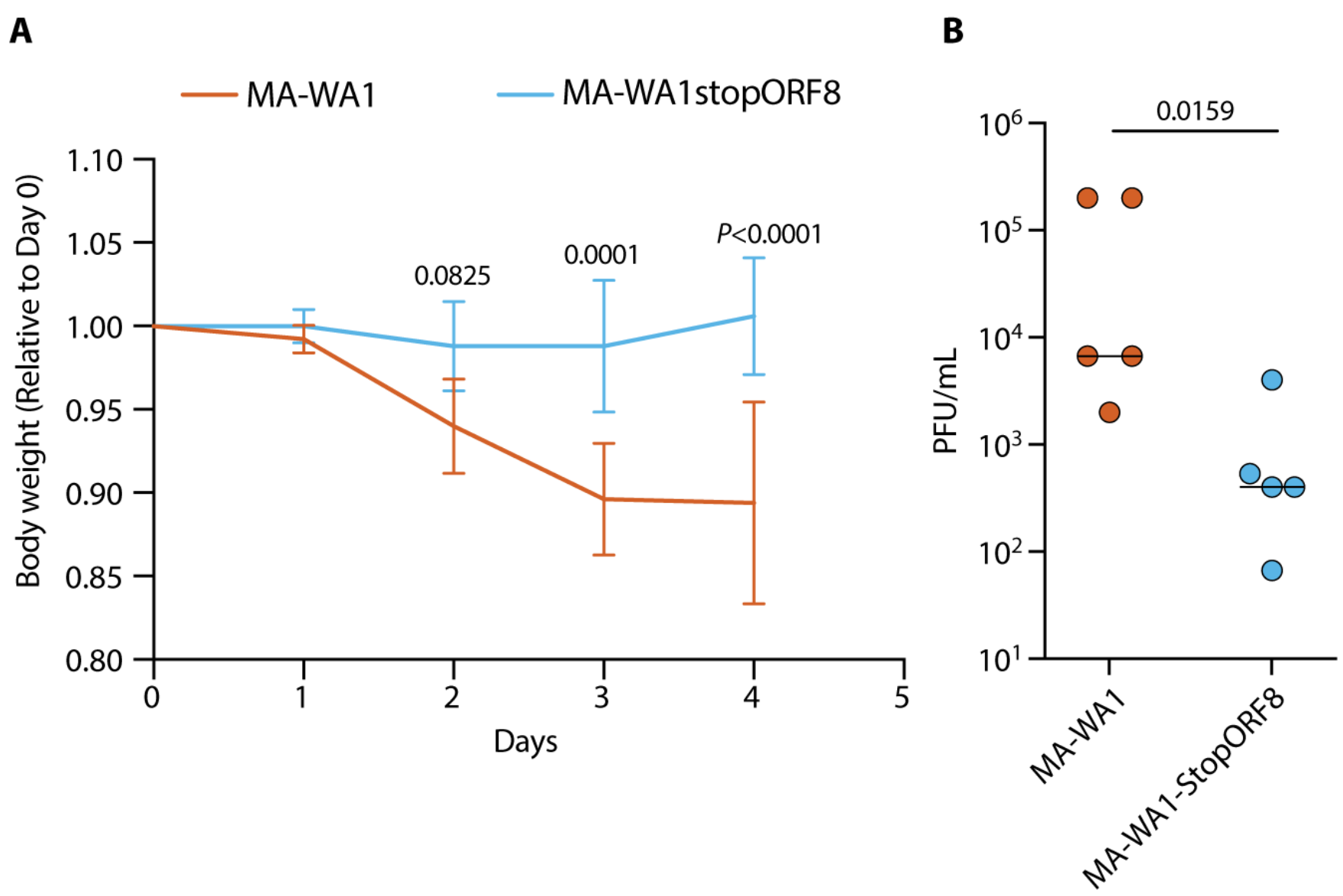

Figure S4. ORF8 determines disease outcome in aged mice.

(A and B) One-year-old male BALB/c mice were intranasally infected with mouse-adapted WA-1 (n = 5) or mouse-adapted WA-1-StopORF8 (n = 5). Body weight was monitored daily (A), and lungs were harvested at 4 days post-infection for plaque assay analysis (B). Statistical significance in (A) was determined by two-way ANOVA with Šídák's multiple-comparisons test, whereas (B) was analyzed using a two-tailed Welch's *t* test.

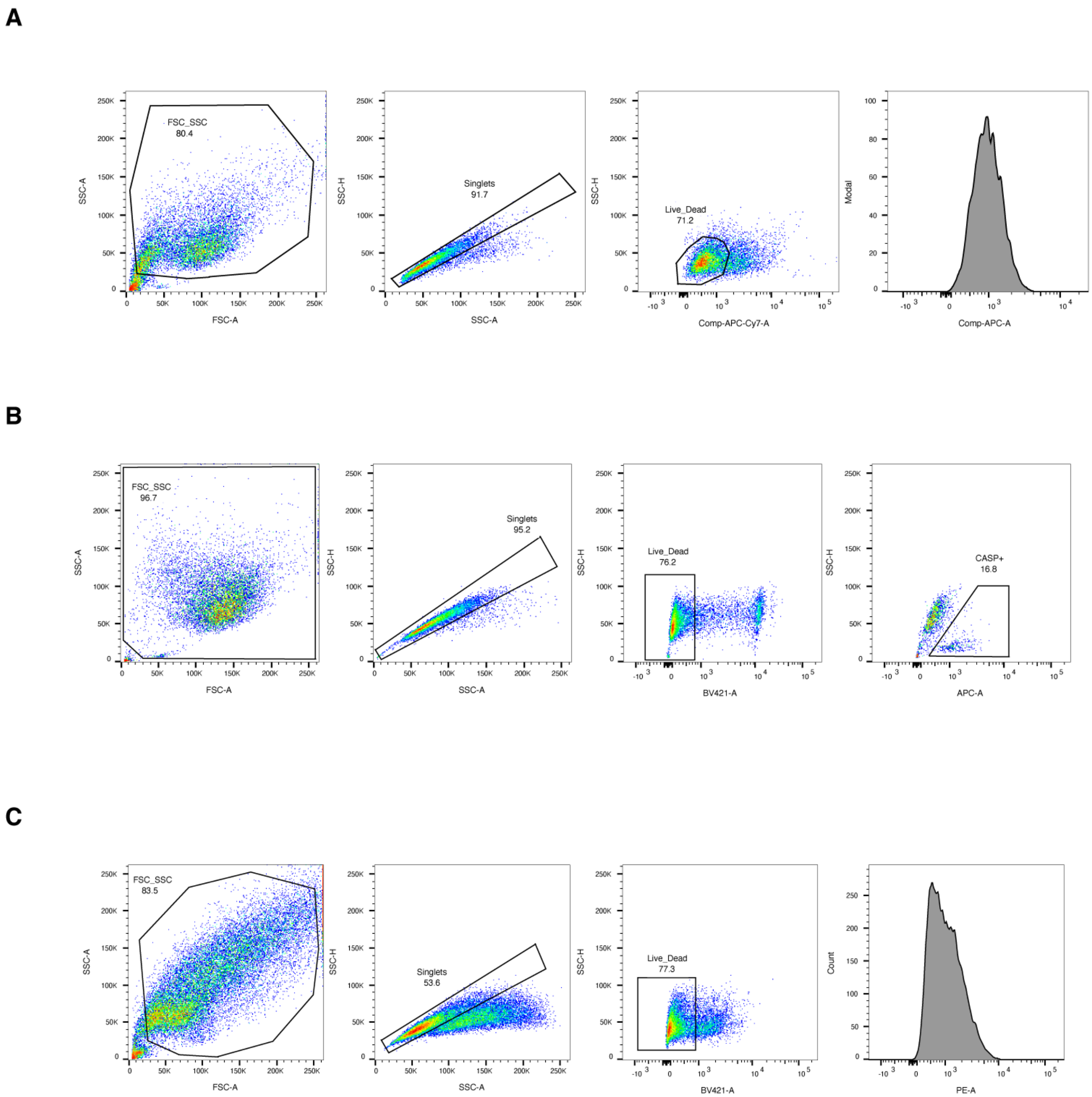

Figure S5. Flow cytometry gating strategy.

(A) Gating strategy for ACE2 expression. (B) Gating strategy for FLICA analysis. (C) Gating strategy for TMPRSS2 expression

Table S1. MSstats quantification of proteins in lysates from M0, M1, and M2 primary monocyte-derived macrophages (MDMs) exposed to ORF8 for 12 and 48 h.
Table S2. Gene Ontology (GO) enrichment analysis of proteins identified in M0, M1, and M2 primary MDMs exposed to ORF8 for 12 and 48 hours.
Table S3. qPCR primer sequences.
Table S4. Metadata overview for the proteomic abundance dataset from M0, M1, and M2 primary MDMs exposed to ORF8.
Table S5. Liquid Chromatography (LC) parameters used for proteomic data acquisition.
Table S6. Orbitrap Exploris 480 mass spectrometry (MS) parameters used for collection of proteomic data acquisition.
Table S7. FragPipe parameters used for proteomic database searching.
